# British Society for Rheumatology guideline on management of adult and juvenile onset Sjögren disease

**DOI:** 10.1093/rheumatology/keae152

**Published:** 2024-04-16

**Authors:** Elizabeth J Price, Stuart Benjamin, Michele Bombardieri, Simon Bowman, Sara Carty, Coziana Ciurtin, Bridget Crampton, Annabel Dawson, Benjamin A Fisher, Ian Giles, Peter Glennon, Monica Gupta, Katie L Hackett, Genevieve Larkin, Wan-Fai Ng, Athimalaipet V Ramanan, Saad Rassam, Saaeha Rauz, Guy Smith, Nurhan Sutcliffe, Anwar Tappuni, Stephen B Walsh

**Affiliations:** Department of Rheumatology, Great Western Hospital NHS Foundation Trust, Swindon, UK; The Academy Library and Information Service, Great Western Hospital NHS Foundation Trust, Swindon, UK; Department of Rheumatology, Barts and The London School of Medicine and Dentistry, Barts Health NHS Trust, London, UK; Centre for Experimental Medicine and Rheumatology, The William Harvey Research Institute, Queen Mary University of London, London, UK; Department of Rheumatology, Milton Keynes University Hospital, Milton Keynes, UK; Department of Rheumatology, University Hospitals Birmingham NHSFT, Birmingham, UK; Institute of Inflammation and Ageing, University of Birmingham, Birmingham, UK; Department of Rheumatology, Great Western Hospital NHS Foundation Trust, Swindon, UK; Centre for Rheumatology, Division of Medicine, University College London, London, UK; Patient Representative, Sjogren’s UK Helpline Lead, Sjogren’s UK (British Sjögren’s Syndrome Association), Birmingham, UK; Patient Representative, Sjogren’s UK (British Sjögren’s Syndrome Association), Birmingham, UK; Rheumatology Research Group, Institute of Inflammation and Ageing, University of Birmingham, Birmingham, UK; National Institute for Health Research (NIHR) Birmingham Biomedical Research Centre and Department of Rheumatology, University Hospitals Birmingham NHS Foundation Trust, Birmingham, UK; Centre for Rheumatology, Division of Medicine, University College London, London, UK; General Practice, NHS Staffordshire & Stoke on Trent ICB, Stafford, UK; Department of Rheumatology, Gartnavel General Hospital, Glasgow, UK; Department of Social Work, Education and Community Wellbeing, Northumbria University, Newcastle upon Tyne, UK; Department of Ophthalmology, Kings College Hospital, London, UK; Translational and Clinical Research Institute & Newcastle NIHR Biomedical Research Centre, Newcastle University, Newcastle upon Tyne, UK; Department of Rheumatology, Newcastle upon Tyne NHS Foundation Trust, Newcastle upon Tyne, UK; Department of Paediatric Rheumatology, Bristol Royal Hospital for Children, Bristol, UK; Translational Health Sciences, University of Bristol, Bristol, UK; Haematology and Haemato-Oncology, KIMS Hospital, Maidstone, Kent, UK; Ophthalmology, Institute of Inflammation and Ageing, University of Birmingham, Birmingham, UK; Birmingham and Midland Eye Centre, Sandwell and West Birmingham NHS Trust, Birmingham, UK; Department of Ophthalmology, Great Western Hospital NHS Foundation Trust, Swindon, UK; Department of Rheumatology, Barts Health NHS Trust, London, UK; Institute of Dentistry, Queen Mary University of London, London, UK; London Tubular Centre, University College London, London, UK

**Keywords:** Sjögren disease, Sjögren’s syndrome, connective tissue disease, guideline, treatment, recommendations, management

## Abstract

Sjögren disease (SD) is a chronic, autoimmune disease of unknown aetiology with significant impact on quality of life. Although dryness (sicca) of the eyes and mouth are the classically described features, dryness of other mucosal surfaces and systemic manifestations are common. The key management aim should be to empower the individual to manage their condition—conserving, replacing and stimulating secretions; and preventing damage and suppressing systemic disease activity. This guideline builds on and widens the recommendations developed for the first guideline published in 2017. We have included advice on the management of children and adolescents where appropriate to provide a comprehensive guideline for UK-based rheumatology teams.

## Graphical abstract

**Graphical Abstract:**
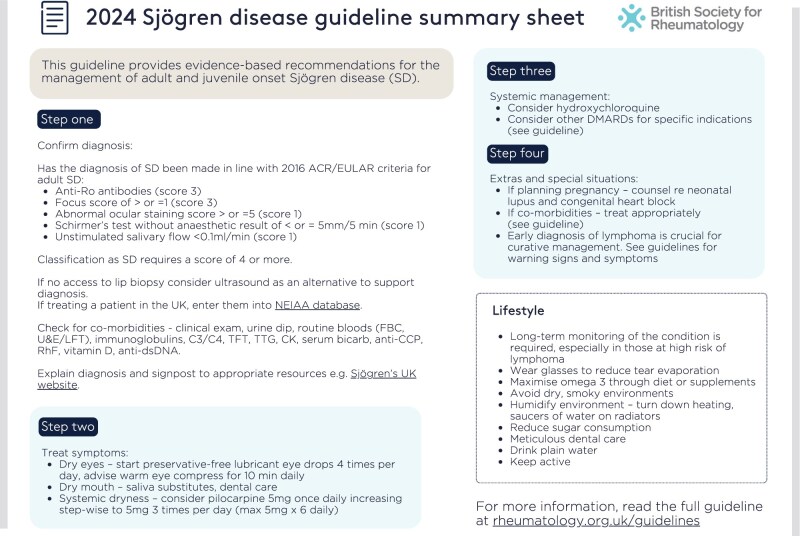


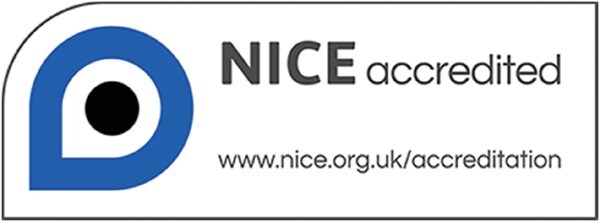


NICE has accredited the process used by BSR to create its clinical guidelines. The term began on 27 February 2012 and the current renewed accreditation is valid until 31 December 2023. More information on accreditation can be viewed at www.nice.org.uk/accreditation.

## Background and rationale for guideline development

The rationale behind this update of the 2017 British Society for Rheumatology (BSR) guideline for the management of Sjögren disease (SD) [1] is described in the guideline scope [2]. SD continues to be a chronic, autoimmune disease of unknown aetiology for which there is no known curative treatment. People with SD report ongoing frustration with the paucity of treatment and the lack of provision and knowledge in the healthcare system [3]. Successful management requires personalization of care. Although dryness (sicca) of the eyes and mouth are the classically described features, dryness of other mucosal surfaces and systemic manifestations, including fatigue and arthralgia, are common. Systemic (extraglandular) features affect at least 70% and include inflammatory arthritis, skin involvement, haematological abnormalities, neuropathies, interstitial lung disease (ILD) and B-cell lymphoma (5–10% lifetime risk) [4, 5]. The key management aim should be to empower the individual to manage their condition—conserving, replacing and stimulating secretions; and preventing damage and suppressing underlying systemic disease activity.

SD has a significant impact on the quality of life (QoL) of affected people. A recent literature review found that health-related QoL (HRQoL) was markedly reduced in SD in multiple studies across many countries when compared with healthy controls [6]. The reduction in HRQoL was similar to that observed in other chronic diseases such as RA and SLE, suggesting that it is not a ‘benign’ disease. This reduction in QoL has been noted in multiple domains and across all populations studied worldwide. Anxiety, depression, pain and fatigue are all increased in SD compared with healthy controls and significantly impact on the QoL [7]. The loss of taste and smell that accompanies SD has a negative effect on the QoL [8] as does the ocular dryness [9, 10]. There is a significant reduction in sexual QOL [11] due to the combined effects of vaginal dryness [12], atrophy [13] and psychosocial factors such as coping strategies and illness perceptions [14]. Systemic involvement, including nervous system manifestations such as peripheral neuropathy [15], respiratory system involvement [16] and arthralgia [17], also have a negative impact on QoL.

Meta-analysis suggests an increase in cardiovascular [18] and respiratory [19] morbidity and a small excess mortality has been observed in people with SD [20], particularly in males and those with underlying lung disease [21]. SD remains a chronic illness with no disease modifying or curative treatments available to date. People can accumulate morbidity over time.

SD may occur alone, when it has traditionally been referred to as primary SD, or alongside another rheumatic disease, when it may present as either an overlap or secondary phenomenon. The ACR/EULAR criteria [22] are now widely used to classify people with primary SD and are often used diagnostically.

This guideline builds on, and widens the recommendations developed for the first guideline published in 2017 [1]. We have included advice on the management of children and adolescents with SD where appropriate to provide as comprehensive a guideline as possible for UK based rheumatology teams.

## Target audience

The target audience includes clinicians caring for individuals with SD and those not satisfying criteria but who present with sicca symptoms. This will include (but is not limited to) paediatric and adult rheumatologists, general practitioners, ophthalmologists, oral medicine specialists, dentists, opticians, optometrists and other clinicians including specialist nurses, Allied Health Professionals and people with SD.

## Guideline development, search methodology and dates

This guideline was developed in line with the BSR Creating Guidelines protocol using AGREE II (Appraisal of guidelines for Research and Evaluation II) methodology. The working group had previously agreed the guideline scope and identified 19 key questions [2]. Using these key questions as a basis a literature search was undertaken in a number of databases (see Supplementary Data S1, available at *Rheumatology* online). We restricted the search to human, English language and the date range 1 January 1990 to 1 December 2022. The eligible papers were reviewed, and draft recommendations developed. The original key questions were expanded where necessary to cover the breadth of the literature. The SIGN (Scottish Intercollegiate Guidelines Network) and GRADE (Grading of Recommendations, Assessment, Development, and Evaluations) processes were used to summarize the quality of the body of evidence for each recommendation as high (A), moderate (B) or low/very low (C) according to GRADE methodology. We have combined C (low) with D (very low) for the purposes of this guideline. Please note that C will include expert consensus where we could find no evidence within the literature.

Where there was no new evidence since the last guideline this is stated. The scope for this guideline is broader than that of the previous guideline. We have looked at additional facets of management and included children and adolescents in our recommendations. In developing the full guideline the lead author drafted the text and circulated it to the whole group. Suggested revisions were incorporated and the revised text circulated multiple times. Where there was disagreement a discussion was commenced via e-mail until a consensus could be reached. We also had two online teams meetings to debate and discuss various points. All authors agreed the final draft before submission.

The content, wording, strength of recommendation (strong = 1, conditional = 2) and Strength of Agreement (SOA) were determined by the working group responses. Only recommendations with a SOA >80% were included.

## Key questions identified in the scope

### 1. In people suspected of having SD, what is the diagnostic accuracy of ANA, ENA and other novel antigen testing?

A total of 518 publications were identified in the initial clinical evidence review for this section. Following initial screening 417 records were removed. This left 101 full-text articles of which a further 81 were excluded for a variety of reasons. The remaining 20 studies were included in the meta-analysis for this section.

Given the evolution of the classification criteria over time direct comparisons between publications can be difficult.

Six studies were identified exploring the diagnostic accuracy of ANA in SD, all but one included a wider population than suspected SD [23–28]. See Table 1 for details. Five of these studies were retrospective cohort studies. The quality of the studies were graded from very low to moderate. Overall, these studies estimated the sensitivity of ANA as between 58% and 85% and the specificity as between 50% and 97%. The only study that confined itself to people with suspected SD (all had sicca) and scored moderate on GRADE found a sensitivity of 85% and specificity of 50% [24]. Median ages for subjects in these studies ranged from 39 to 60 years. No correlation was reported between age and ANA positivity.

**Table 1.** Summary of evidence on diagnostic accuracy of ANA in SD and various CTDs

Study	Population	Diagnosis	Index tests	Ref. standard	Comments	Sensitivity % and specificity % (95% CI) of ENA in CTD	AURC (95% CI)
Jeong *et al*. (2018) [23]	*N* = 1115; suspected of AARD; of whom 19 were diagnosed with SD	Various AARDs	ANA—indirect immunofluorescence	Expert clinical diagnosis using AECC criteria	Retrospective cohort study; conducted in 2 hospitals in Korea	Sensitivity 58% (33–80%); specificity 80% (77–82%)	Not reported
Santiago *et al*. (2015) [24]	*N* = 218; all had sicca	SD	ANA—indirect immunofluorescence	Minor salivary gland biopsy	Prospective cohort study at single hospital in Argentina	Sensitivity 84% (75–92%); specificity 50% (42–59%)	Not reported
Ulvestad (2001) [25]	*N* = 446; unselected rheumatology patients, 4 of whom were diagnosed with SD	Rheumatology patient cohort	ANA—ELISA and indirect immunofluorescence	Preliminary European criteria	Retrospective cohort study; Norway	Indirect immunofluorescence: sensitivity 73% (54–88%); specificity 96% (93–97%); ELISA: sensitivity 63% (44–80%); specificity 96% (77–82%)	
Ulvestad (2003) [26]	*N* = 407; unselected rheumatology patients, 73 of whom were diagnosed with SD	Rheumatology patient cohort	ANA—indirect immunofluorescence	Preliminary European criteria	Retrospective cohort study; Norway	Indirect immunofluorescence: sensitivity 63% (51–73%); specificity 76% (71–80%)	AURC 0.865
Willems *et al*. (2018) [27]	*N* = 9856; consecutive ANA tests; 63 later diagnosed with SD	Consecutive ANA tests; unselected	ANA—indirect immunofluorescence and FEIA	ACR classification criteria	Retrospective cohort study; Belgium	Results reported as AURC: indirect immunofluorescence: 0.803 (0.799–0.892); FEIA: 0.924 (0.876–0.971)	AURC values is in the column to the left
Zafrir *et al*. (2013) [28]	*N* = 242; 67 healthy controls, 107 PBC; 20 scleroderma, 48 SD	Selected population of CTD and healthy controls in a single centre	ANA—indirect immunofluorescence	ACR classification criteria	Retrospective cohort study, Tel Aviv	Sensitivity 65% (49–78%); specificity 97% (90–100%)	

The area under (a receiver operating characteristic) curve is a measure of the accuracy of a quantitative diagnostic test. A test with no better accuracy than chance has an AUC of 0.5, a test with perfect accuracy has an AUC of 1 (DeLong ER, DeLong DM, Clarke-Pearson DL. Comparing the areas under two or more correlated receiver operating characteristic curves: a nonparametric approach. Biometrics 1988;44:837–45). AUC can be misleading as it gives equal weight to the full range of sensitivity and specificity values even though a limited range, or specific threshold, may be of practical interest (Hanley JA, McNeil BJ. The meaning and use of the area under a receiver operating characteristic (ROC) curve. Radiology 1982;143:29–36). AARD: antibody- associated rheumatic disease; AECC: American–European Consensus Classification; AURC: area under the curve; FEIA: fluorenzyme immunoassay; PBC: primary biliary cholangitis.

Three studies were identified exploring the diagnostic accuracy of ENA—although none was specific for SD [23, 29, 30]. See Table 2 for details. The studies were graded as low quality due to risk of bias. Results showed that the estimated sensitivity for ENA ranged between 89% and 92%; with a specificity of 71–77%. In a very small number of cases individuals can be ANA negative but Ro positive [31].

**Table 2.** Summary of evidence on diagnostic accuracy of ENA in SD

Study	Population	Diagnosis	Index Tests	Ref standard	Comments	Sensitivity % and Specificity % (95% CI) in CTD	AURC (95% CI)
Bentow *et al*. 2013 [29]	*N* = 1079	Various AARDs including SD	Two tests: 7-test ENA panel (ELISA); 6-test NA panel (ELISA)	Unclear	Prospective cohort study; not specific to Sjogren’s	Sensitivity 92% (79–98%); specificity 74% (71–77%)	0.97 (0.93–1.0)
Jeong 2018 [23]	*N* = 1115	Various AARDs including SD	9-test ENA panel (ELISA)	Expert clinical diagnosis using AECC	Retrospective cohort study	Sensitivity 90% (76–97%); specificity 71% (68–73%)	0.97 (0.94–0.99)
Pi *et al*. 2012 [30]	*N* = 329	Various AARDs including SD	Two tests: 6-test NA panel (ELISA); MPBI	Physician diagnosed—criteria not specified	Retrospective cohort study	Sensitivity 89% (67–99%); specificity 77% (74–79%)	0.94 (0.91–0.98)

The area under (a receiver operating characteristic) curve is a measure of the accuracy of a quantitative diagnostic test. A test with no better accuracy than chance has an AUC of 0.5, a test with perfect accuracy has an AUC of 1 (DeLong ER, DeLong DM, Clarke-Pearson DL. Comparing the areas under two or more correlated receiver operating characteristic curves: a nonparametric approach. Biometrics 1988;44:837–45). AUC can be misleading as it gives equal weight to the full range of sensitivity and specificity values even though a limited range, or specific threshold, may be of practical interest (Hanley JA, McNeil BJ. The meaning and use of the area under a receiver operating characteristic (ROC) curve. Radiology 1982;143:29–36). AARD: antibody-associated rheumatic disease; AECC: American–European Consensus Classification; AURC: area under the curve; MPBI: multiplex bead–based immunoassay.

All three studies reported the sensitivity and specificity of ENA in patients with a variety of underlying CTD including SD. The ENA panels used varied between 6-, 7-, 9- and 14-test ENA panels, and also included one multiplex bead–based immunoassay (MPBI). One [23] study used two different tests which between them included testing for dsDNA, U1RNP, Sm, Ro/SSA60 and 52, La/SSB, Scl-70, Pm-scl, Jo-1, CENP, PCNA, nucleosomes, histones, ribosomal-P and AMA-M2. Only 19 of the patients had clinical SD.

Bentow *et al.* [29] also used two different tests (one six- and the other seven-test panels) and reported the sensitivity and specificity of ENA in patients with a variety of underlying CTD including SD in 39.

Pi *et al.* [30] used a six-test panel and a MPBI assay and reported that SSA and SSB were shown to be the critical determinants for the diagnosis of SD with both immunoassays in the 23 patients studied.

In all the studies the numbers of patients with SD were small and the authors have reported sensitivity and specificity data for ENA overall and not for Ro and/or La or SD specifically.

Positive RF is a common finding in people with SD (48.6% in one large series of >10 000 individuals [32]) and RF IgA and IgG have been suggested as potential biomarkers of SD. In a study of 76 people with SD classified by the 2016 ACR/EULAR criteria, IgA RF was noted to have higher sensitivity than IgM or IgG RF (72% *vs* 61% *vs* 51%) with a strong association noted between IgA RF and the presence of anti-Ro/La antibodies [33]. There was no control population. In another study with a control group (77 with SD and 37 sicca controls) IgA RF was reported to have a sensitivity 83.1% and specificity 78.4% in distinguishing SD from non-SD sicca [34].

A small number of studies were found reporting on the diagnostic accuracy of novel antigen testing in SD, including a metanalysis [35] of the anti-alpha-fodrin antibody test. The meta-analysis reviewed a total of 23 studies all published before the publication of the 2012 ACR criteria and found a pooled sensitivity of 39.3% and specificity 83.1%. The authors concluded that anti-alpha-fodrin testing showed moderate accuracy for the diagnosis of SD with high specificity and relatively low sensitivity. A comparison of the use of early SD autoantibodies (SP1, anti-salivary protein; CA6, anti-carbonic anhydrase VI; PSP, anti-parotid secretory protein) *vs* classical autoantibodies (ANA, anti-Ro/La, RF) found that the early autoantibodies underperformed in comparison to the classical autoantibodies in differentiating sicca from juvenile SD (jSD) [36]. A systematic review of salivary biomarkers in people with SD [37] concluded that salivary autoantibodies were less sensitive than anti Ro/La antibodies. Currently none of the ‘novel’ autoantibodies out-perform anti-Ro antibody and are therefore not recommended outside a research setting.

In summary (and bearing in mind the caveats discussed above):

- ANA—sensitivity 58–85%; specificity 50–97%
- ENA—sensitivity 89–92%; specificity 71–77%
- IgA RF—sensitivity 72–83.1% and specificity 78.4%
- Novel antigens—anti-alpha-fodrin antibody sensitivity 39.3%, specificity 83.1%; early SD autoantibodies (SP1, anti-salivary protein; CA6, anti-carbonic anhydrase VI; PSP, anti-parotid secretory protein) sensitivity 55.6%, specificity 26.9%

ANA is commonly used as a screening antibody in clinical scenarios where SD or other CTDs are suspected. Because of its frequency and low specificity, it should not be measured in the absence of clinical indicators of SD or other CTD. If there is a high suspicion of SD an ENA should be tested even if the ANA is negative.

### Recommendation

Do not measure ANA in the absence of clinical indicators of SD or other CTD (1, C) (SOA 94.6%).

Use ANA as a screening antibody where there is clinical suspicion of a CTD (1, C) (SOA 93.9%).

Measure ENA even if the ANA is negative if there is a high index of suspicion of SD (1, C) (SOA 96.7%).

### 2a. In people suspected of having SD, what is the diagnostic accuracy of salivary gland US scanning?

In 2017 an atlas of the most common parenchymal abnormalities seen on US scanning (USS) was published by the US-pSS Study group [38] and in 2019 the OMERACT USS working group developed a consensus salivary gland US score [39]. They described a novel four-grade semi-quantitative scoring system for the parotid and submandibular glands ranging from grade 0—normal, through to grade 3—severe changes, and showed that adding USS to the 2016 ACR/EULAR criteria improved sensitivity from 90.2% to 95.6% [40]. Following the publication of these criteria a meta-analysis of 65 studies published in 2020 [41], which included 54 diagnostic accuracy studies and a total of 6087 individuals, plus two more recent accuracy studies involving 269 and 243 individuals [42, 43] all confirmed the utility of USS in the diagnosis of SD. Overall sensitivity in the meta-analysis was 80% with a specificity of 90%. The two additional studies were consistent with this, reporting a sensitivity of 69% and 72% and specificity of 98% and 94%, respectively.

A worldwide cohort study in jSD found pathologic USS changes in 61% of individuals, which correlated with hyposalivation, autoantibody seropositivity and a history of glandular swelling [44], whilst a single-centre study reported USS changes in 96% [45]. These studies support the use of USS as an additional diagnostic tool in young people who often have little or no dryness and therefore do not fulfil the adult classification criteria.

In ENA-negative individuals especially, USS performed by an expert is useful to aid diagnosis. USS is also safe and useful if salivary gland biopsy is not available or not possible (e.g. in individuals on anticoagulation where it is unsafe to stop) and may be helpful to differentiate other causes of sicca symptoms and glandular enlargement. A caveat is that USS may not be able to differentiate between SD and sarcoid or other CTDs [45] and many of the diagnostic studies did not include other disease controls. Studies from tertiary centres have shown that if both serology is negative and USS is normal then the pick-up rate on salivary gland biopsy is low [42].

USS of the salivary glands can provide useful confirmatory information to support either the presence of or lack of evidence for SD but does not currently form part of the classification criteria. However, there is accumulating evidence of good correlation between USS abnormalities and positive biopsies [46] with a single-centre study of 103 consecutive individuals showing good agreement between USS and parotid (83%) and labial (79%) biopsies and good predictive value. A high correlation has been confirmed between the salivary gland USS score and the focus score in individuals participating in the multicentre TEARS study [47].

### Recommendation

USS of the salivary glands can provide useful additional information to support either the presence of or lack of evidence for SD (1, A) (SOA 95.2%).

USS does not currently replace either antibody testing or histological analysis in adult SD classification criteria (1, A) (SOA 96.4%).

### 2b. In people suspected of having SD, what is the diagnostic accuracy of other imaging modalities?

There is a smaller evidence base for other imaging modalities including CT, PET and MRI. A single-centre study of 34 people with SD, 22 with sicca and 57 asymptomatic controls confirmed that parotid CT was accurate and reliable in differentiating those with SD from both sicca and normal controls [48]. A small study of 23 people with SD and 23 healthy controls found that dual protocol MRI scanning of the lacrimal glands achieved a sensitivity of 92% and specificity of 83% [49]. PET scanning has been shown to be helpful in the detection and management of lymphoma in SD [50]. Reviews of imaging modalities in SD [51, 52] have concluded that further larger studies are needed to establish the role of PET, CT and MRI in diagnosis and monitoring of SD. None of the imaging modalities is included in the most recent classification criteria [22].

### Recommendation

Overall, although they may provide useful supplementary information, we do not recommend additional imaging modalities over and above USS in the routine assessment of SD (1, C) (SOA 97.3%).

### 3a. In people suspected of having SD, what is the diagnostic accuracy of major and minor salivary gland biopsy?

Six suitable studies were identified looking at the diagnostic accuracy of labial salivary gland biopsy [53–58]. These reported a sensitivity of 80–92% and specificity of 88–97%.

One case series of 50 individuals described minor complications of labial salivary gland biopsy in up to 20% (6% transient sensory defect, 6% transient local pain, 2% transient localized burning, 6% cutaneous haematoma and 4% mild mucosal inflammation) [59]. In another larger retrospective study of 630 individuals across two centres, 20% reported long standing impairment of sensation post-biopsy although with a low level of impact on everyday life [60]. A third study involving 186 individuals reported loss of sensation in only 3% [61]. A systematic review comparing complication rates in those undergoing a minimally invasive technique compared with a linear incision technique found the pooled prevalence of permanent neurological adverse events was eight times lower in the minimally invasive group (0.17% *vs* 1.45%) [62]. A recently published study has confirmed the safety in a case series of 110 people undergoing biopsy [63]. Only four experienced temporary lip numbness with no permanent complications.

Consensus guidelines on reporting of labial salivary gland biopsy have been developed by the EULAR Sjogren’s Syndrome (EULAR SS) study group and recommend reporting the Focus Score (i.e. number of foci of >50 mononuclear cells per 4 mm^2^ of tissue) [64].

One study directly compared parotid to labial salivary gland biopsy [65]. All 110 underwent simultaneous parotid and labial salivary gland biopsies. At 1 week and 6 months post-procedure they reported more pain and numbness in the parotid biopsy site but by 12 months symptoms were minor and comparable at both sites. A recent single-centre study in 29 individuals has investigated US-guided core needle biopsy of parotid glands and reported adequate samples for diagnosis in 96.5% of cases [66].

There is evidence that if the serology and salivary gland USS results are compatible (e.g. both negative or both positive), then a biopsy is of little added value [41, 42]. There is increasing evidence that the diagnosis of SD can be confirmed or excluded without a biopsy [67], although the current classification criteria do not include USS [22].

Minor labial salivary gland biopsy can also provide additional prognostic data regarding lymphoma risk in both seronegative and seropositive individuals, and this is discussed in more detail below.

The Childhood Arthritis and Rheumatology Research Alliance (CARRA) survey showed that 51% of clinicians performed labial salivary gland biopsy for diagnostic purposes in children presenting with probable SD. In an international cohort study published by the group biopsy information was available on 131 (44%) of the 300 cases [68]. A recent cohort study of 39 children from China reported the use of diagnostic labial salivary gland biopsy in 97.4% [69].

Parotid gland biopsy also facilitated SD diagnosis in a small case series of children with jSD with negative labial gland biopsy [70], whilst in those with lacrimal gland inflammation, lacrimal biopsy identified SD as the most common diagnosis [71], suggesting that parotid and lacrimal biopsies can be used in selected cases.

In summary:

- Minor (labial) SG biopsy—sensitivity 80–92%; specificity 88–97%—forms an essential part of the most recent 2016 ACR/EULAR classification criteria when individuals are anti-Ro antibody negative and there is objective evidence of sicca affecting eyes and mouth [22]
- Complication rates of minor salivary gland biopsy are low overall and lower in those undergoing minimally invasive technique compared with a linear incision technique [54]
- Parotid gland biopsy—sensitivity 78%; specificity 86% [54]. Complication rates of parotid gland biopsy were low with no permanent sensory loss observed in one small case series [54] and similar rates to labial salivary gland biopsy in another [65]
- A recent small study has investigated US-guided core needle biopsy of parotid glands and reported adequate samples for diagnosis in 97% of cases [66]. It is not widely available in the UK and further larger studies may be required to understand the reliability and comparability of this approach compared with conventional approaches

### Recommendation

Consider a minor labial salivary gland biopsy to aid diagnosis in those with clinically suspected SD where the diagnosis cannot be made by clinical and serological features alone (1, A) (SOA 98.2%).

### 3b. In people suspected of having SD, what is the diagnostic accuracy of lacrimal gland biopsy?

Studies of lacrimal glands in SD show characteristic patterns of inflammation with clusters of predominantly CD8+ T lymphocytes around acinar epithelial cells which may be driving the secretory dysfunction. A single-centre retrospective study of 60 individuals presenting with features suggestive of lacrimal inflammation (i.e. erythema, oedema or tenderness) showed diagnostic features of SD or other identifiable conditions in 37 (61.7%) [71].

### Recommendation

There is currently insufficient evidence to routinely recommend lacrimal gland biopsy in SD (1, C) (SOA 98.2%).

### 4a. In people with confirmed SD are there any measurable biomarkers that can predict development of lymphoma?

The evidence review for biomarkers identified 493 potential studies, of which 461 were excluded on screening. Thirty-two full texts were assessed for eligibility, 28 excluded and 4 studies selected for meta-analysis.

A case–control study of 381 primary SD without and 92 primary SD with concomitant non-Hodgkin lymphoma (NHL) found seven factors to be independent predictors for future lymphoma [72]:

- Salivary gland enlargement
- Lymphadenopathy
- RP
- Anti-Ro and/or La autoantibodies
- RF
- Monoclonal gammopathy
- Low complement component 4 (C4) most predictive

The presence of two or fewer of these seven factors resulted in a 3.8% probability for the later development of lymphoma; three to six factors in a 39.9% probability; and if all seven, then 100% of this patient group developed lymphoma.

In another reported single-centre study 11 of a cohort of 244 developed an NHL [73]. In this study purpura, parotid enlargement, anaemia, leucopaenia, lymphocytopaenia, hypergammaglobulinemia, low C3 and low C4 were all found to be significant predictors of NHL, but only hypocomplementaemia and lymphocytopaenia were independent risk factors. In an earlier retrospective study by Baimpa *et al.* of 536 consecutive individuals with SD, 7.5% developed lymphoma [74]. The development of NHL in this cohort was predicted by the presence of neutropaenia (*P* = 0.041), cryoglobulinaemia (*P* = 0.008), splenomegaly (*P* = 0.006), lymphadenopathy (*P* = 0.021) and low C4 levels (*P* = 0.009). Individuals with any of these factors had a 5-fold increased risk.

Ioannidis *et al.* [75] performed predictive modelling in a cohort of 723 consecutive individuals with SD and found that the probability of lymphoproliferative disease (LPD) was 2.6% at 5 years and 3.9% at 10 years. LPD was independently predicted by the presence of parotid enlargement [hazard ratio (HR) 5.21], palpable purpura (HR 4.16) and low C4 (HR 2.40) at first study visit.

Brito-Zerón *et al.* [76] retrospectively looked at 1300 cases of SD and found that after a median follow-up of 66.1 months (range 1–560.3 months; 9922.3 person-years), 127 (9.8%) developed 133 cancers: 64 developed a solid cancer, 57 an haematological cancer, 4 developed both solid and haematological cancers, and 2 developed two different types of solid neoplasia [10]. The most frequent types of cancers included B cell mucosa–associated lymphoid tissue (MALT) lymphomas (*n* = 27, 20%) and other B cell NHL (*n* = 19, 14%). Those who developed MALT NHL had a higher frequency at diagnosis of cryoglobulins (*P* = 0.002), low C3 levels (*P* = 0.018), high EULAR SS disease activity index (ESSDAI) score of 4 or more (*P* = 0.001), and high joint DAS score (*P* < 0.001), while the risk of non-MALT B cell lymphomas was unrelated to systemic activity, with anaemia, monoclonal gammopathy, cryoglobulins and low C4 levels at SD diagnosis being the main risk factors.

There were similar findings in a multicentre case–control study including 101 individuals with SD and lymphoma where salivary gland enlargement, the presence of RF, low C4, cryoglobulinemia, lymphopaenia and disease activity as measured by ESSDAI were all found to be predictors of lymphoma in the multivariate analysis [77].

Some retrospective studies have suggested a link between the presence of germinal centres, focus score and future lymphoma development. Theander *et al.* [78] reviewed the salivary gland biopsies of 175 individuals with SD and identified lymphoid organization in the form of germinal centres in 25% at diagnosis. Seven developed lymphoma during follow-up, of whom six had germinal centres at diagnosis. However, this finding was not confirmed in a subsequent very small study which reviewed the biopsies of 11 individuals who had developed lymphoma and compared these with SD controls who had not developed lymphoma, and found similar low rates of germinal centre formation in both groups [79]. Risselada *et al.* [80], in a retrospective analysis of 174 individuals with primary SD, reported that the threshold of three or more foci had a positive predictive value of 16% for lymphoma and a negative predictive value of 98%. A link between focus score and lymphoma was reported in a retrospective review of 794 individuals with SD, of whom 34 developed lymphomas during follow-up [81]. A more recent study has proposed salivary gland focus score as a biomarker for lymphoma development [82]. The authors found that focus score at diagnosis, cryoglobulinaemia and salivary gland enlargement were independent risk factors for the future development of lymphoma. Those with a focus score ≥4 had a statistically significant shorter time interval from SD to lymphoma diagnosis than those with a focus score <4 (4 *vs* 9 years).

Goules *et al.* [83] looked at the influence of age of onset of SD on later lymphoma development. They identified a cohort of 379 individuals with age of onset <35 years and compared these with 293 with age of onset >65 years. They found that in the younger age of onset group cryoglobulinemia, C4 hypocomplementemia, lymphadenopathy and salivary gland enlargement were independent lymphoma associated factors, whereas in the older age of onset group salivary gland enlargement, C4 hypocomplementemia and male gender were the independent lymphoma associated factors. Early onset individuals displayed two incidence peaks of lymphoma within 3 years of onset and after 10 years, while in late onset group, lymphoma occurred within the first 6 years.

In children with jSD, MALT lymphomas have been described as initial presentation or associated with recurrent parotitis, lymphadenopathy and presence of auto-antibodies [84]

From these studies, and acknowledging the differences in case ascertainment and other factors, the following consistently emerge as predictors of future lymphoma development:

- Low C3/C4 with low C4 being the strongest predictor
- Clinical evidence of salivary gland enlargement
- Clinical evidence of lymphadenopathy
- Cryoglobulinaemia
- Monoclonal gammopathy
- High focus score (>4)

In addition, clinical signs and symptoms associated with lymphoma should alert to the possible existence of early/microscopic NHL. These include B symptoms (persistent night sweats, fevers and weight loss of ≥10% over the preceding 3 months), clonal lymphocytosis in peripheral blood flow cytometry and splenomegaly.

### Recommendation

Individuals with SD should be offered further investigation early if they present with new salivary gland swelling or other symptoms that might suggest the development of lymphoma (1, A) (SOA 98.75%).

Consider a minor labial salivary gland biopsy to provide additional prognostic data regarding lymphoma risk in both seronegative and seropositive individuals (2, C) (SOA 92.7%).

### 4b. In people with confirmed SD are there any measurable biomarkers that can predict disease progression or development of extraglandular disease?

Although SD is characterized by ocular and oral dryness systemic manifestations are common and include joint, skin, lung, cardiac, gastrointestinal and nervous system involvement [85]. There are similarities and overlaps with the predictive markers for lymphoma with a number of features being associated with the development of extraglandular disease.

A single-centre cross-sectional study involving 64 individuals [86] found that ANA was associated with younger age of onset and renal involvement [risk ratio (RR) 1.25]. Anti-Ro was associated with younger age, renal involvement (RR 1.36) and high ESSDAI. Anti-La was positively associated with renal involvement (RR 3.4) and negatively with articular involvement (RR 2.75). RF was associated with haematological involvement and hypergammaglobulinemia was associated with younger age of onset.

RF positivity has been associated with an increased prevalence of systemic disease in a number of studies (reviewed in [87]). A retrospective review of 275 individuals with SD confirmed an association between persistent serological disease activity and the presence of a positive RF [88].

There have been attempts at stratifying people with SD into high and low risk for the development of systemic disease and progression. One proposal suggests classifying groups into low, moderate and high risk of progression based on the following phenotypes [89]:

- Low risk—elderly onset, seronegative, isolated anti-La antibody positive
- Moderate risk—Black/African American, young onset, anti-Ro antibody positive
- High risk—males, high focus score or germinal centre formation, RF positive, cryoglobulinaemia, hypocomplementaemia

In summary the following features are associated with a higher risk of progression to systemic extraglandular disease:

- Anti-Ro antibody positive
- Younger age of onset
- Ethnicity (Black/African American)
- Males
- RF positive

### Recommendation

Baseline assessment of individuals with SD should include a thorough clinical and serological evaluation to inform the risk of development of extraglandular features and disease progression (1, B) (SOA 97.6%).

### 5. In people with confirmed SD what other investigations should routinely be undertaken to exclude common associated conditions, for example coeliac or thyroid disease?

Comorbidities are common in SD. In a population based series of individuals with SD identified via health insurance records the most frequent reported comorbid conditions were hypertension, OA, osteoporosis and depression [90] with 22% having co-existent thyroid disease [90]. In the UK Primary Sjogren’s Syndrome Registry (UKPSSR) cohort comorbidities increased with age and BMI and the most common were OA (36%), gastro-oesophageal reflux (31%), hypertension (20%), chronic cystitis (10%), hypercholesterolaemia (10%), asthma (9%), osteoporosis (8%), FM (8%), irritable bowel syndrome (8%) and ischaemic heart disease (5%) [91].

Given that hypertension is a modifiable risk factor for the development of myocardial infarction and stroke we would recommend pro-active treatment if this were identified. EULAR recommendations have been developed for the management of cardiovascular risk in people with rheumatic diseases [92]. They make recommendations for SD including the use of population-based prediction tools (e.g. QRISK3), blood pressure and lipid management as per population recommendations. They advise platelet inhibition only as per general population recommendations.

In a population-based study in Norway [93] the authors looked at nearly 13 000 adults—they performed Tissue Transglutaminase (TTG) IgA testing and proceeded to offer duodenal biopsy to those that tested positive. They found that 1.47% of the population had coeliac disease of whom 75% were previously undiagnosed. Furthermore, switching to a gluten free diet resulted in significant improvement in gastrointestinal symptoms and HRQoL.

Evidence of coeliac disease was found in 4.5% of those with SD in one Hungarian study [94]. This compares with a prevalence of 4.5–5.5 per 1000 in the normal European population. In another European study antibodies to TTG, an antibody strongly associated with coeliac disease, were present in 12% of those with SD compared with 4% of normal controls. On further investigation over 70% of the anti-TTG positive individuals were found to have biopsy evidence of coeliac disease [95]. Overall therefore coeliac disease is 10 times more common in SD than in the normal population.

Mild elevation of liver enzymes may be seen in SD but most of these individuals are asymptomatic and more serious liver disease is rare. In an observational study of 300 individuals with primary SD some signs of liver involvement were found in 7% but the majority of these were asymptomatic [96]. Data from the UKPSSR [97] showed that, amongst 549 subjects where an extensive auto-antibody profile was available, only 0.9% were positive for anti-mitochondrial antibody (AMA) and all of these were also positive for anti-Ro and/or anti-La antibodies [98]. The most common associated autoimmune liver condition is primary biliary cholangitis (PBC) with co-existent SD reported in 3.5—36% of patients with PBC [99–102], with the lowest rates in the European studies and the highest rates in a Chinese population. Conversely PBC has been found in 4–9% of those with SD in studies of European and American populations [103–106].

The risk of acute pancreatitis was found to be increased in SD compared with the general population (HR 1.48, 95% CI 1.03–2.12) in a large, population based study in Taiwan [107].

Monoclonal gammopathy was detected in 22% of a European cohort of 221 individuals with primary SD [108]. In this cohort monoclonal gammopathy was associated with a higher prevalence of parotid enlargement, extraglandular features, hypergammaglobulinaemia, cryoglobulinaema, RF and hypocomplementaemia. A systematic review investigating the link between monoclonal gammopathy and autoimmune rheumatic disease found that those with SD were at highest risk of developing a monoclonal gammopathy with an odds ratio of 4.51 [109].

Distal renal tubular acidosis (dRTA), secondary to a chronic tubulointerstitial nephritis, is associated with SD and may be complete (with a systemic acidosis) or incomplete (urinary acidification defect without acidosis). The estimated prevalence of complete dRTA is 5% and of incomplete 25% [110, 111]. A low serum bicarbonate is compatible with complete dRTA. More complex testing may be required if the dRTA is incomplete [112]. The tubulointerstitial nephritis and other renal manifestations, such as immune complex glomerulonephritis (mesangioproliferative glomerulonephritis, usually associated with lymphomatous transformation) can cause significant renal impairment [113].

Compared with adults, children with jSD had more frequent neurologic and renal manifestations [114].

Muscle pain (myalgia) is common in primary SD but objective evidence of myositis is much less common. Anecdotal case reports and small case series are reported in the literature [115, 116].

A large multicentre cohort [117] reviewed 1320 individuals with primary SD and found muscular weakness in only 17 (1.28%). Nearly half of this small group (41.1%) had myalgia, 76.4% had an increased creatine-phosphokinase (CPK) and an abnormal EMG was found in 13 out of the 14 where it was tested (92.8%). Of the 13 undergoing muscle biopsy, 6 were found to have histological evidence of myositis giving an incidence of histologically proven myositis of just 0.45%.

Inclusion body myositis has been described in small numbers of individuals with SD [116, 118, 119]. Usual age of presentation for this group was in their 50s. The prevalence at ∼0.6% is possibly higher than the background population prevalence which is estimated at 3.5/100 000, with the condition being more common in males (3:1) and usually presenting at >50 years [120]. Data from the prospective ASSESS cohort published in 2021 found a prevalence of 0.5% [121], which is higher than that reported in unselected populations [122].

Vitamin D deficiency is common at latitudes >40 degrees from the equator with up to 30% of adults in the UK having low vitamin D levels in the winter months [123]. An association has been noted between low vitamin D levels, peripheral neuropathy and lymphoma in SD [124]. A systematic review and meta-analysis of vitamin D deficiency and severity of dry-eye symptoms in SD [125] included a total of 18 studies and concluded that overall individuals with vitamin D deficiency had shorter tear break-up time (TBUT), lower Schirmer’s scores and higher Ocular Surface Disease Index (OSDI—a patient reported outcome measure). In addition vitamin D levels were found to be lower in SD than controls.

### Recommendation

Be aware of and consider screening for commonly associated conditions, as guided by age and/or clinical presentation (1, B) (SOA 94.7%).

We recommend that the following additional investigations are undertaken at baseline, and repeated as clinically indicated, to detect comorbidities and associated autoimmune diseases:

- Vitamin D levels; (1, B) (SOA 95.6%)
- Thyroid function
- Liver function tests (and anti-mitochondrial antibodies if indicated)
- TTG
- Immunoglobulins and serum electrophoresis
- Serum bicarbonate
- Creatine Kinase

### 6. In people with SD who have sicca (dryness) symptoms of the eyes, what is the most clinically effective topical treatment?

A total of 1083 studies dealing with topical treatments or dry eyes were identified as part of this systematic review; 1008 excluded after initial screening and 75 full-text articles assessed for eligibility. Of these, 49 were excluded for a variety of reasons (see Supplementary Data S1, available at *Rheumatology* online). Twenty-six were included in the final analysis [12 primary studies including 11 randomized controlled trials (RCT) and one non-randomized study (NRS) in SD; 14 systematic reviews in a wider dry-eye population]. Much of the evidence is based on studies looking at the dry-eye population in general, with very few looking exclusively at SD-related dry eye.

In addition to the formal literature review it was felt important to highlight that SD is associated with complex eye disease [126] with aqueous tear deficiency, meibomian gland dysfunction [127, 128] and surface inflammation contributing to the symptom load. Frequently, symptoms outweigh the signs. Effective management addresses the aqueous and meibomian gland deficiency and treats any surface inflammation. Some individuals have corneal neuropathic pain that does not resolve with these treatments.

Lifestyle measures should also be considered. Low humidity speeds up evaporation of tears and where possible individuals should avoid overheated and air-conditioned environments. Relative humidity has a significant effect upon dry-eye symptoms [129] and the UK Health and Safety Executive (UKHSE) recommend workplace relative humidity should be between 40% and 70%. Dry eyes start to be a problem even for healthy workers below 20%.

The frequency of instillation of eye drops is also important—with evidence suggesting that 2–3 hourly is optimum [130].

#### Lubricating eye drops

A Cochrane review of lubricating drops for dry eye included 43 RCTs of 3497 participants with dry eye [131]. Lack of concordance between the inclusion criteria and measurements limited the ability to undertake a full meta-analysis. They concluded that lubricating eye drops were generally safe with similar efficacy, but that inconsistencies in trial design and reporting led to a high risk of bias and made comparisons difficult. They did find that lubricating eye drops as a whole consistently improved ocular symptoms. The most common adverse events were blurred vision, ocular discomfort and foreign body sensation. The design of the studies and lack of comparators made it difficult to identify any individual formulation as being superior to others.

A recently published systematic review [132] identified 64 relevant articles and concluded that there is good evidence that lubricating eye drops improve symptoms of dry-eye disease within a month of regular use but that signs of dry-eye disease take longer to improve. They concluded that individuals should be offered non-preserved or soft preserved eye drops to avoid worsening of the dry-eye disease due to the toxic, proinflammatory and detergent effects of the preservative; those with evaporative dry eyes should be prescribed a formulation with a high concentration of liposomes; and that individuals should be advised to use their drops at least four times daily for at least a month before reassessment. They found some evidence that drops containing polyethylene glycol were more effective than those containing carboxymethylcellulose/carmellose sodium and hydroxypropyl methylcellulose and that combination formulations were more effective than single active ingredient lubricating eye drops.

A meta-analysis of the efficacy of hyaluronic acid (HA) eye drops for dry eye, not specific to SD, included 17 studies (12 parallel and 5 crossover, all randomized) and 1339 cases [133]. They found some evidence that HA eye drops were superior to saline or non-HA-based drops. There was a significant increase in Schirmer’s test values in the HA group overall and a significant increase in TBUT compared with saline eyedrops. Data on fluorescein staining was available in four studies with no evidence that HA eye drops were superior to non-HA based drops. Data on OSDI were available in five studies, with a tendency towards decreased symptoms in the HA-treated group, but this failed to reach significance.

A systematic review of lubricating eye drops [132] concluded that sodium hyaluronate combined with carboxymethylcellulose was more effective than either in isolation, that HA and sodium hyaluronate benefited from the addition of trehalose and that Coenzyme Q10 enhanced the effectiveness of HA.

In general, most of us treating patients with SD associated dry eye would start with a sodium hyaluronate–containing drop during the day and an eye ointment at night; the carmellose-based drops may offer better retention and polyvinyl alcohol containing or combination drops containing lipids are beneficial in stabilizing the tear film. Always prescribe preservative free drops. Be aware that formulations change frequently and some drops become unavailable at short notice. Be prepared to substitute formulations if needed ensuring you always prescribe a preservative free option.

### Recommendation

Advise regular use of a preservative free lubricating eye drop (e.g. 2–3 hourly) (1, A) (SOA 94.4%).

#### Serum eye drops

Blood-derived eye drops may be autologous, i.e. prepared from an individual’s own peripheral blood (such as autologous serum, platelet-rich plasma and platelet lysate) or allogeneic, i.e. prepared from donors (such as allogeneic peripheral blood serum and umbilical cord blood serum). A pilot study comparing the two types found comparable efficacy and tolerability [134]. A systematic review and meta-analysis of serum eyedrops for dry eye included 19 studies involving 729 participants [135]. Of these, 10 compared autologous serum to lubricating eye drops. There was a trend towards improvement in OSDI and TBUT in those treated with autologous serum eyedrops but no difference in Schirmer’s testing or fluorescein staining between the groups.

A more recent systematic review and meta-analysis of autologous serum eyedrops for dry eye included a total of seven RCTs with 267 subjects [136]. There was statistically significant evidence of improvement in OSDI, TBUT and Rose Bengal staining score in those treated with autologous serum eyedrops compared with lubricating eye drops with those receiving autologous serum eyedrops reporting better symptom relief. There was no difference in Schirmer’s testing or fluorescein staining between the groups.

In the UK serum eye drops are only available via specialized centres in line with published National Health Service (NHS) policy.

### Recommendation

Autologous or allogeneic serum eye drops may be offered to individuals with ongoing symptoms despite maximal management with conventional eye drops (1, A) (SOA 91.9%).

Note that in the UK serum eye drops are only available via specialized centres in line with published NHS policy.

#### Topical steroid eye drops

A Cochrane review of topical CS for dry-eye disease identified 22 RCTs (4169 participants) conducted worldwide [137]. Overall, they found a small to moderate improvement in patient reported symptoms as compared with lubricants alone; a small to moderate improvement in corneal staining score; a slight increase in TBUT but no change in tear osmolality. They concluded that for dry eye requiring anti-inflammatory control, topical steroids provided a small to moderate degree of symptom relief beyond lubricants.

A review of 16 studies looking at loteprednol etabonate (LE) steroid eye drops (14 prospective, 2 retrospective) found that treatment with LE reduced signs of inflammation without clinically significant intra-ocular pressure elevation [138]. Additionally, pre-treatment with LE reduced stinging upon subsequent ciclosporin instillation.

A randomized clinical trial of topical fluorometholone 0.1% eyedrops *vs* ciclosporin 0.05% eye drops in 40 individuals with SD-associated dry eye found that both treatments reduced corneal fluorescein staining, patient-reported OSDI and increased conjunctival goblet cell density after 8 weeks of therapy [139]. Onset of action was faster in the fluorometholone group with benefit at 4 weeks but no significant difference between the groups at 8 weeks.

### Recommendation

Topical steroid eye drops, under ophthalmic supervision, may be offered short term to individuals with ongoing persistent inflammation despite maximal management with conventional eye drops (1, A) (SOA 94.9%).

#### Immunomodulating eye drops

##### Ciclosporin

A systematic literature review of the use of topical immunomodulatory drugs including ciclosporin, diquafosol, lifitegrast and rebamipide included 26 trials [140] of which 24 were RCTs, and found inconsistencies in reported outcomes. Significant improvements in dryness were reported in one study of ciclosporin emulsion, but not in two others. In three studies involving those with aqueous dry eye of differing cause, corneal staining and Schirmer’s scores were significantly improved in the ciclosporin group, with one study also demonstrating significant improvement in the TBUT. Improvements were less marked in the studies involving those with evaporative dry eye. Ciclosporin eye drops can also be used off-label in children and adolescents from 4 years of age, based on the efficacy observed in keratoconjunctivitis [141], but there are no published studies in jSD.

##### Tacrolimus

0.1% tacrolimus eye drops have been evaluated in a small number of individuals with severe allergic conjunctival disease [142] and been shown to be safe and effective for this indication. Topical 0.03% tacrolimus eye drops were evaluated in eight individuals with dry eye in an open label study in a single centre. There were statistically significant improvements in fluorescein and Rose Bengal staining and TBUT, but no improvement in Schirmer’s testing over the 90 days of treatment [143]. Topical tacrolimus 0.03% has been evaluated alongside ciclosporin 0.05% in a cohort of 60 individuals with SD where each acted as their own control by using the active eye drop in one eye and a placebo in the other [144]. Both active ingredients significantly improved symptoms, reduced frequency of lubricating eye drop use and ocular staining compared with the placebo controlled eye with no significant difference between the groups. Tacrolimus eye drops are not currently routinely available in the UK.

##### Topical IL-1 antagonist

Proof of concept studies [145] have shown a significant improvement in OSDI. However, these are early phase studies intended as proof of concept only and the preparation is not currently commercially available.

##### Lifitegrast

Lifitegrast is a topical lymphocyte function associated antigen 1 antagonist (LFA-1 antagonist) approved in the USA in 2016 but not currently European Union approved. There have been four large multicentre RCTs (results summarized in [140]) which showed a significant improvement in inferior corneal staining score and a visual analogue score (VAS) measure of eye dryness. Lifitegrast is licensed in the USA and Far East but not currently National Institute for Health and Care Excellence (NICE)-approved nor available in the UK (https://www.nice.org.uk/guidance/indevelopment/gid-ta10196).

##### Rebamipide

Rebamipide eye drops—a quinolone derivative—increases corneal and conjunctival mucin levels [146] and have been shown to stabilize the tear film [147] in a small prospective randomized study in 20 individuals. It has been available in Japan since 2012 but is not currently available in UK or Europe, nor is it NICE-approved within the UK.

##### Diquafosol

Diquafosol eye drops are available as a 3% ophthalmic solution. Diquafosol is a purinergic P2Y2 receptor agonist which promotes fluid transfer and mucin secretions by activating P2Y receptors on the ocular surface. Meta-analysis of nine RCTs [148] showed significant improvements in Schirmer’s test, fluorescein staining and TBUT compared with control. Diquafosol is available in Japan but not currently available in UK/Europe. Diquafosol eye drops are also recommended for use in jSD based on data from the adult studies in the Japanese guidelines [149].

### Recommendation

Topical ciclosporin eye drops, under ophthalmic supervision, may be indicated for those with persistent surface inflammation despite maximal management with conventional eye drops (1, B) (SOA 94.9%).

#### Treatments for meibomian gland deficiency

A systematic review of evidence based treatments for meibomian gland deficiency (MGD) found 35 relevant articles and found that all eight standard forms of treatment including, self-applied eyelid warming, thermal pulsation, IPL, MG probing, antibiotics, lipid containing eye drops and perfluorohexyloctane, were effective against MGD, although with varying extent of clinical improvements [150].

##### Warm compresses

A systematic review of treatments for MGD [150] found eight studies (five RCTs and three NRS) looking at the use of a reusable warm compress. All eight demonstrated efficacy in achieving clinical improvements in symptoms and tear film metrics.

#### Recommendation

Advise a heated eyelid compress for at least 10 min daily (1, A) (SOA 94.9%).

##### Lipiflow (thermal pulsation)

Thermal pulsation (lipiflow) therapy—four studies (two RCTs, two NRS) [150]. Single session sufficient to produce improvement in the OSDI score and meibomian gland secretion score.

Only currently available in the UK via private providers—not NHS funded.

##### Intense pulsed light therapy

A 2020 Cochrane review of intense pulsed light (IPL) in the treatment of meibomian gland disease [151] looked at three RCTs and concluded that conclusive evidence of efficacy was not available.

Subsequently a 2021 systematic review of IPL therapy for MGD, not specific to SD, found nine studies with a total of 539 individuals [152]. They concluded that IPL combined with meibomian gland expression (MGX) may be a safe and effective treatment for MGD but IPL alone was not superior to MGX alone. IPL is only currently available in the UK via private providers and is not NHS funded.

##### Meibomian gland probing

A systematic review of meibomian gland probing, antibiotics, lipid-containing eye drops and perfluorohexyloctane found that all were effective against MGD, although with varying extent of clinical improvements [150].

Meibomian gland probing is performed as an in-office procedure, under slit lamp guidance using a fine probe (∼80 µm wide and 2 mm long) and an initial description of the procedure in 2010 [153] has led to a flurry of reports of its efficacy. A critical evaluation of the literature on meibomian gland probing published in 2020 reviewed 14 studies of which four were RCTs. Numbers per study ranged from 3 to 49. Results varied—most showed an improvement, but the controlled studies failed to show a significant difference between groups. The procedure seemed most effective in combination with other treatments such as IPL and repeated treatments were often needed. Meibomian gland probing did not consistently out-perform standard care nor was it better than the placebo effect of sham probing.

Only currently available in the UK via private providers—not NHS funded.

#### Recommendation

Lipiflow, IPL therapy and meibomian gland probing are not currently NHS funded as treatments within the UK. There is currently insufficient evidence to recommend their routine use. However, these procedures are safe with, in some cases, weak evidence of benefit in dry eye and individuals may decide to undergo these treatments in the private sector (2, C) (SOA 84.5%).

#### Antibiotics for meibomian gland disease

A recent review of antibiotic treatment for dry-eye disease with meibomian gland dysfunction or blepharitis included 22 articles [154]. The authors concluded that both oral and topical antibiotic treatment resulted in short-term improvements but noted that improvements were not sustained when treatment was discontinued and felt there was insufficient evidence to recommend long-term use.

#### Recommendation

Those with dry-eye disease associated with meibomian gland dysfunction or blepharitis could be offered short-term treatment with oral or topical antibiotics with an anti-inflammatory action (2, B) (SOA 92.3%).

#### Lipid-containing eye drops

Lipid-containing eye drops have been shown to be effective in MGD [150]. A systematic review of lipid containing lubricants published in 2012 included three studies on liposomal eye sprays and four on lipid-containing eyedrops [155]. None of the studies was free of bias and only three were double masked. All subjects reported symptomatic improvement although this was short-lived in two studies. TBUT improved in four of the five studies where it was measured. Three studies were assigned high level of evidence, three moderate and one low.

#### Recommendation

Individuals with dry-eye disease associated with meibomian gland dysfunction or blepharitis could be advised to use lipid containing eye drops or liposomal eye sprays as adjunctive treatment (2, C) (SOA 90.2%).

#### Punctal occlusion

A Cochrane review [156] included 18 trials and 711 participants. Overall, they concluded that the evidence of benefit was inconclusive although individual studies suggest that punctal plugs may improve symptoms.

Expert opinion is that punctal plugs are suitable in certain circumstances but they may make corneal surface inflammation worse in certain situations. Careful patient selection is important.

#### Recommendation

Punctal plugs are suitable in in certain circumstances, but they may make corneal surface inflammation worse in certain situations. Careful patient selection is important (1, C) SOA 96.3%.

#### Androgen replacement therapy

A systematic review of seven studies looked at the role of androgen-replacement therapy in dry-eye disease [157]. All studies were small (10–62 individuals) and most included those with dry-eye disease of varying aetiology. Three were RCTs. Five used androgen-replacement ointments containing 1–5% testosterone applied topically to skin. One study investigated the use of oral DHEA (dehydroepiandrosterone, a testosterone precursor) capsules and the final study investigated the use of a DHEA-containing eye drop. Six showed a benefit over a short (2–4 weeks) study period. One (in solely SD) showed no benefit. All studies were too short to assess long term benefits.

#### Recommendation

There is insufficient evidence to recommend androgen replacement therapy for dry-eye disease (2 C) (SOA 96.3%).

### 7. In people with SD who have sicca (dryness) symptoms of the mouth, what is the most clinically effective topical treatment?

A Cochrane review [158] of topical treatments for dry mouth of any cause (including SD) found no strong evidence supporting one topical therapy over another. The authors reviewed 36 RCTs involving 1597 participants. Two compared saliva stimulants to placebo, nine compared saliva substitutes to placebo, five compared saliva stimulants directly with saliva substitutes, 18 directly compared two or more saliva substitutes, and 2 trials compared two or more saliva stimulants. Oxygenated glyceroltriester saliva substitute spray showed evidence of improved effectiveness compared with an electrolyte spray (standardized mean difference 0.77, 95% CI 0.38–1.15) which corresponds to approximately a mean difference of 2 points on a 10-point VAS for mouth dryness. Chewing gum was associated with increased saliva production in the majority of those with residual capacity but there was no evidence that gum was more or less effective than saliva substitutes.

A Cochrane review of non-pharmacological therapies for dry mouth [159] including acupuncture (five studies), electrostimulation (three studies) and powered *vs* manual toothbrushing (one study) found low quality evidence that acupuncture is no different from placebo, insufficient evidence on the effect of the electrostimulation device and no evidence of a difference between manual and powered toothbrushing on the symptoms of a dry mouth.

‘Oil pulling’—a technique derived from Ayurvedic medicine—has been proposed as a treatment for dry mouth. It involves rinsing the mouth with coconut or olive oil for about 5–20 min. There is anecdotal evidence of benefit and a study in 2017 showed improvement in subjective symptoms of xerostomia. A small randomized, single-blind, crossover trial in 26 individuals with medication-induced xerostomia showed no difference in rinsing with water compared with oil [160].

### Recommendation

Suggest saliva substitutes for symptomatic relief of oral dryness (2, C) (SOA 93.3%).

### 8. In people with SD who have sicca (dryness) symptoms outside the eyes and mouth, what is the most clinically effective topical treatment?

#### Topical treatments for vaginal dryness

Vaginal dryness is a common symptom in SD. One study recorded self-reported vaginal dryness in 53% compared with 28% of controls *P* = 0.005 [161]. Despite this there are no published studies of treatment of vaginal dryness specifically in SD.

A Cochrane review of topical oestrogens [162] for vaginal atrophy in post-menopausal women included 30 RCTs (6235 women) and found low to moderate quality evidence of benefit *vs* placebo. There was no difference in efficacy between the various intravaginal preparations.

Topical oestrogen use is regarded as safe and no association was found between vaginal oestrogen use and multiple health outcomes including cardiovascular disease, cancers and hip fracture in a cohort of nearly 900 women participating in the Nurses’ Health Study—a large population based cohort involving >50 000 individuals studied over 18 years of follow-up [163]. Topical oestrogens are not recommended for use in children or adolescents.

Non-hormonal vaginal moisturizers have been shown to provide effective symptomatic relief of vaginal dryness in normal post-menopausal women [164–166] and are routinely recommended in guidelines [166, 167]. They are available over the counter. Two studies [164, 165] found vaginal HA to be as effective as vaginal estriol in post-menopausal women (not SD) for the treatment of vaginal dryness and associated symptoms of itching, burning and dyspareunia.

#### Recommendations

Consider advising topical non-hormonal vaginal moisturizers plus oestrogen creams/pessaries in peri- or post-menopausal women with significant vaginal dryness (2, C) (SOA 97.5%).

### 9a. In people with SD who have sicca (dryness) symptoms, what is the most clinically effective stimulatory treatment?

#### Stimulatory treatments for ocular sicca

There are no recent studies of pilocarpine in SD, but some good evidence of benefit from historical studies. A double blind RCT of pilocarpine 20–30 mg daily from 2004 involving 256 individuals with SD showed significant improvement in global assessment of dry eye and relief in six of eight related symptoms at 12 weeks (global improvement in dry eye, improved eye comfort, reduced foreign body sensation, decreased use of tear substitutes, reduced light sensitivity, reduced matting and sticking).

A smaller unblinded RCT (*N* = 85) [168] of pilocarpine 5 mg bd showed improvement in symptom VAS and Rose Bengal staining (but no significant change in Schirmer’s).

A large (*N* = 373) double blind RCT of pilocarpine 10 mg or 20 mg daily [169]. Those in the 20 mg group demonstrated significant improvement in global symptoms of dry eye.

There is anecdotal evidence that starting with a low dose and titrating upwards over time reduces side effects.

Two double-blind RCTs have compared cevimeline to placebo for the treatment of dry eye [170, 171]. There was weak evidence of a clinical benefit to cevimeline—although this is currently not available in the UK or Europe and is not licensed for children.

#### Recommendation

Consider a trial of pilocarpine (5 mg once daily increasing to 5 mg tds/qds) in those with significant ocular sicca symptoms with evidence of residual glandular function (1, A) (SOA 95.3%).

#### Stimulatory treatments for oral sicca

Two large RCT’s including 629 individuals with SD [169, 172] confirmed significant improvement in oral dryness and salivary flow rates with pilocarpine but side effects were common—sweating 43%, urinary frequency 10% and flushing 10%. Three RCTs [170, 173, 174] confirmed improved oral dryness and salivary flow rates for cevimiline but with a high frequency of sweating and nausea. Cevimeline is not available in the UK or Europe and is not licensed for children. Only one comparative study was identified [175], suggesting similar efficacy but cevimeline better tolerated with less severe sweating (11% *vs* 25%) and lower failure rates as a consequence.

#### Recommendation

Consider a trial of pilocarpine (5 mg once daily increasing to 5 mg tds/qds) in those with significant oral sicca symptoms with evidence of residual glandular function (1, A) (SOA 98.4%).

### 9b. What is the clinical effectiveness of fluoride, xylitol, chlorhexidine, artificial saliva or diet in preventing the development or progression of dental caries and gum disease?

None of the published evidence is SD specific and much of the evidence is old. Most of the evidence relates to children and adolescents, with little evidence in adults. A 2019 Cochrane review to determine the influence of fluoride toothpaste on caries prevention concluded that fluoride toothpaste was more beneficial in caries prevention than no-fluoride toothpaste, with a dose–response effect noted in children and adolescents [176]. Evidence on the efficacy of higher dose fluoride toothpastes is limited [176]. The maximum concentration of fluoride-containing toothpaste that can be purchased over the counter in the UK is 1500 p.p.m. fluoride. Higher concentrations are available on prescription from a dentist and Public Health England allow this for those susceptible to dental caries who are unable to reduce their susceptibility over time [177]. A 2015 Cochrane review supports the use of xylitol in caries prevention in children. It works by reducing *Streptococcus mutans* carriage [178]. A Cochrane review of chlorhexidine to prevent dental caries in children and adolescents included eight RCTs for chlorhexidine (varnishes/gels)—not SD specific, mostly done in children—found little evidence of benefit over placebo [179]. A Cochrane review of water fluoridation for caries prevention found very little contemporary evidence of benefit [180]. Studies from pre-1975 indicated that water fluoridation is effective at reducing caries in permanent dentition in children. Fluoride varnishes were confirmed to have a substantial caries-inhibiting effect in children and adolescents in a Cochrane review [181]. Interdental cleaning is important in reducing gingivitis and plaque and contributes to caries prevention; interdental brushes may be more effective than flossing [182].

There is evidence from the historical literature that frequency of sugar intake is important in the development of dental decay but no new studies [183].

### Recommendation

Recommend regular brushing with fluoride toothpaste, proactive dental care and the use of xylitol containing products as an alternative to sugar to prevent dental decay (2, C) (SOA 95.6%).

### 10a. In people with SD what is the clinical effectiveness of treatments in comparison to each other or placebo for treating systemic disease?

Systemic (extraglandular) features are seen in up to 70% of individuals with SD and are severe in 15% [184]. Most involved organs are joints, lungs, skin and peripheral nerves [5]. Raynaud’s and thyroid disease tend to be more common in females and lung involvement and peripheral neuropathies are more common in those with disease duration of >10 years [4]. Other systemic features may include autoimmune liver disease, renal involvement, subacute cutaneous lupus, immune thrombocytopaenia, myositis, monoclonal gammopathy of uncertain significance and lymphoma [185]. There is increasing recognition of neuropsychiatric symptoms [186].

#### Conventional immunomodulatory drugs

##### Hydroxychloroquine

There are a number of studies involving HCQ [187–194], but no new studies since the last guideline was published. The largest (JOQUER) did not reach its primary outcome but there was a trend to improved joint pain on long-term follow-up [191]. In addition, reanalysis of the trial by stratifying individuals into different symptom-based subgroups, revealed that those with high symptom burden showed significant improvements in the ESSPRI score [195, 196]. A recent systematic review and meta-analysis [197] of the use of HCQ in SD included 13 studies and 987 individuals with SD (9 from the English literature and 4 published in Chinese). The authors concluded that HCQ showed significant efficacy in improving oral symptoms, unstimulated salivary flow rates, inflammatory indices and immunoglobulins, but not ocular symptoms, fatigue or extraglandular manifestations. However, the reviewers combined RCTs, observational studies and single-arm studies where they had used the control as baseline. This is likely to have biased the results. HCQ can be used off-label in children from the age of 2 years.

Indirect evidence of the benefit of HCQ is provided by the KISS cohort study [198] and by a multicentre retrospective study from Argentina [199]. The KISS cohort followed 256 individuals with SD over three years. They found that the use of HCQ was associated with less solid organ damage (*P* = 0.008) over the 3-year follow-up period. In the Argentinian cohort which included 221 individuals, of whom 77% were exposed to HCQ, they found a lower prevalence of arthritis, fatigue, purpura, Raynaud’s and hypergammaglobulinemia in the HCQ-treated group.

#### Recommendation

In those with significant fatigue and systemic symptoms consider a trial of HCQ for 6–12 months (2, C) (SOA 95.6%).

#### Corticosteroids

There are case reports and small case series suggesting that CS (e.g. prednisolone and prednisone) help certain systemic features including lung disease [200–202], cytopaenias [203], and, in combination with CYC, neurological involvement [204, 205]. A small open-label study of low dose prednisolone (5–7.5 mg per day) in just 20 individuals in a single centre showed improvements in sicca symptoms and modest improvements in salivary flow [206]. The North American and European guidelines recommend short-term CS use if required but in general urge the use of steroid-sparing agents if use continues [1, 207, 208]. There is no good evidence of benefit in general, but steroids remain widely used for specific systemic manifestations, including renal involvement [114], and as short courses for parotid swelling [209].

#### Recommendation

Systemic steroids may be used short term for specific indications but should not be offered routinely in the management of SD (2, C) (SOA 97.7%).

#### Treatment of systemic disease – conventional immunosuppressive drugs

The evidence base for the use of immunosuppressive drugs other than HCQ in SD is poor and individual practice varies considerably. We summarize the available evidence below but would recommend that any decisions on the use of immunosuppressive drugs are made on a case-by-case basis.

Aside from HCQ there have been a number of relatively low-quality studies looking at the use of other immunosuppressives [210–216]. All were small, mostly not RCT and most showed no benefit.

AZA has been reported as helpful in case reports for systemic complications such as lung disease [217], myelopathy [211] and cytopaenias [218], but an RCT suggested that it did not have a routine role in treatment and was associated with a high frequency of side effects [210]. The Japanese guideline [219] did not recommend AZA and other guidelines suggest it only when other treatment strategies have failed or where a steroid-sparing effect is required.

MTX is considered the drug of choice for people with RA and significant inflammatory arthritis associated with SD [220]. An open-label, pilot study of weekly MTX in 17 individuals with SD showed improvement in sicca symptoms, parotid swelling, dry cough and purpura, but no improvement in objective parameters of dry eyes and mouth [212]. Despite the lack of clear evidence of efficacy and paucity of trial data, the European and North American guidelines all recommend the use of MTX in SD-associated joint disease [1, 207, 208, 221].

A single-centre, open-label trial of mycophenolate in just 11 individuals reported a significant reduction in hypergammaglobulinemia and an increase in complement levels, but little effect on glandular features [214]. Case reports [222] support the use of mycophenolate in SD-associated agranulocytosis and ILD [223, 224]. It is not recommended in the Japanese or the North American guidelines [219] but Saraux *et al.* [221] suggest considering it for lung disease, and there is evidence for a role in the management of CTD-associated ILD [225].

There was some benefit in a LEF alone study in SD involving only 15 individuals of whom most developed significant side effects [213]. A more recent RCT of 29 individuals on LEF/HCQ combination therapy did show some clinical benefit with a significant decrease in ESSDAI score and little in the way of side effects [216, 226], and is supported by immunological evidence of benefit [226]. Further studies of combination therapy are planned.

There are anecdotal reports of successful treatment of SD-associated interstitial cystitis [227], annular erythema [228, 229], red cell aplasia [229] and pneumonitis [231] with oral ciclosporin. An open-label phase II study of low dose ciclosporin A (2 mg/kg) showed reductions in joint swelling and tenderness [232]. The Japanese guidelines do not recommend it [219] and the North American guidelines found scant evidence for its use [208].

There are no controlled trials of CYC in SD and in general its potential toxicity would preclude routine use. However there are published case reports and series documenting successful treatment of SD-associated myelopathy [204, 233], refractory thrombocytopaenia [234], glomerulonephritis [235, 236] and lung disease [237]. In practice, its use is reserved for those with progressive organ-threatening disease and in many of these clinical situations, rituximab would now be the treatment of choice across North America and Europe. The Japanese guidelines suggests its use in those with lung, kidney or CNS involvement [219].

Most of the conventional immunosuppressive drugs can be used off-label in children from the age of 2 years, with the exception of LEF which is not approved for use in people younger than 18 years.

#### Recommendation

Conventional immunosuppressive drugs are not routinely recommended for use in SD outside of the treatment of specific systemic complications (2, C) (SOA 94.7%).

#### Treatment of systemic disease – biologic drugs

Biologics are not NICE-approved for SD. Of the few patients who do get biologics this is usually either as part of a clinical trial or because they meet criteria for RA or another CTD (usually SLE).

All of the recent RCTs in SD rely on ESSDAI for their primary endpoint. There are significant limitations to ESSDAI and in light of this two new outcome measure, CRESS [238] and STAR [239], have been developed and proposed for future use. Reanalysis of some of the studies shown below using CRESS has shown a statistical response to treatment intervention. No studies have been performed in jSD, although biologic therapies can be used off-label for specific indications, e.g. rituximab from 3–6 months, abatacept and anti-TNF agents from 2 years of age and belimumab from 5 years of age.

##### Abatacept

An initial open-label pilot study of abatacept in 11 individuals with primary SD was reported as showing improvement in salivary flow and a reduction in focal glandular inflammation on minor salivary gland biopsy although this was not corrected for background area [240].

A subsequent open label proof of concept study in 15 individuals found that the drug was well tolerated with improvement in fatigue and health related quality of life measures. Despite this there was no change in objective measures of glandular function over a 24-week treatment period [241]. A longer-term open label prospective observational study of 11 individuals on abatacept for 24 months showed small but statistically significant improvements in salivary flow and ESSDAI score but no improvement in fatigue or ocular symptoms or signs [242]. However a recent RCT of abatacept in 80 individuals with SD—the ASAP III study—showed no difference in the primary outcome of between-group difference in ESSDAI score at week 24, leading the authors to conclude that they could not recommend abatacept as treatment for SD [243]. Subsequent reanalysis of ASAP III using CRESS suggested a statistical response to treatment intervention—CRESS response rates at the primary endpoint visits were 60% (24 of 40) for abatacept *vs* 18% (7 of 39) for placebo (*P* < 0.0001) in ASAP III, and 45% (41 of 92) for abatacept *vs* 32% (30 of 95) for placebo (*P* = 0.067) in the multinational abatacept trial. It should be noted that CRESS was developed using data from ASAP III trial and thus some caution should be applied in interpreting the data. Reanalysing the data using the STAR response did not materially change the outcome and there were no changes in most histopathology parameters. Overall, the evidence for abatacept remains inconclusive and more studies are needed before abatacept could be routinely recommended.

##### Anti-IL-1 targeted biologics (anakinra)

A small RCT of 26 individuals [244] found a transient but non-significant reduction in fatigue and concluded that there was no significant benefit overall. A systematic review of the efficacy of anti-IL-1-targeted therapies in the treatment of immune-mediated disease [245] found no further evidence of efficacy for SD.

##### Anti-TNF therapies

Infliximab was initially reported as being beneficial on the basis of two open label studies [246, 247] but both of these apparently positive studies were subsequently retracted because of evidence that methodological errors had led to the wrong conclusions [248]. A small, open-label study of 15 individuals given weekly s.c. etanercept showed no improvement in salivary or glandular function and only 4 of the 15 reported an improvement in fatigue [249].

A number of RCTs were undertaken in light of the initially positive published results from the open-label studies. These failed to show either clinical or serological improvement with etanercept [250] or infliximab [251]. In light of this, none of the recently published guidelines recommends anti-TNF agents as treatment for primary SD although individuals with RA or another CTD can safely receive anti-TNF for their associated disease if needed [207, 208].

##### Baminercept

Baminercept is a lymphotoxin beta receptor IgG fusion protein that blocks lymphotoxin beta receptor signalling. In a multicentre RCT including 52 individuals with SD there was no demonstrable benefit on glandular or extraglandular disease [252].

##### Belimumab

A small open-label study of belimumab in active SD recruited 30 individuals and demonstrated a small improvement in the ESSDAI score from baseline. The effect was most marked in the glandular domain [253]. There are theoretical reasons to support combination use of rituximab and belimumab and some evidence of efficacy in a single reported case [254, 255]. Belimumab has been studied in combination with rituximab, with the latter being used to induce B-cell depletion and belimumab being utilized to maintain the effect, in a phase II double-blind study [256]. A total of 86 individuals were randomized to four treatment arms including placebo. ESSDAI reductions were numerically greater over time with combination treatment than with placebo with almost complete B-cell depletion on minor salivary gland biopsy. The European guidelines have suggested belimumab as rescue therapy in those with severe systemic disease refractory to conventional immunosuppression and rituximab [257].

Recurrent parotid swelling is one of the most common manifestations in children and adolescents. Belimumab may be a potential beneficial treatment for this in selected jSD cases. Although American clinicians have reported the use of both belimumab and abatacept for recurrent parotitis as well as jSD in general [258] there are no published studies or case reports.

##### Epratuzumab

Epratuzumab, a human anti-CD22 monoclonal IgG antibody, was first trialled in an observational study in SD [259]. In this small, open-label study, 16 individuals were enrolled to receive up to four infusions of epratuzumab. Reductions of up to 50% were seen in B-cell levels with just over half achieving a clinical response. Statistically significant improvements were seen in fatigue and patient and physician global assessments. These findings, combined with those seen in open label studies in SLE, led to the phase III EMBODY I and II trials investigating the effects of epratuzumab in moderate to severe SLE [260]. Unfortunately, neither showed a benefit for epratuzumab over placebo despite a documented effect on B-cell populations, with a median reduction of 30–40% in peripheral B-cell levels. A subsequent *post hoc* analysis looked in detail at the 113 individuals who were both anti-Ro positive and had a diagnosis of SD [261]. They noted that this subgroup had a faster reduction in B-cell numbers with evidence of increased B cell sensitivity and a higher proportion showing a lupus clinical response to treatment without an increase in adverse events. SD-related outcomes were not measured. There are currently no ongoing studies of epratuzumab in either SD or SLE.

##### Ianalumab (VAY736)

Iamalumab is a mAb that both depletes B cells and blocks BAFF receptor, thus potentially circumventing the amplified BAFF response seen post-B-cell depletion with other agents such as rituximab. A phase II study in a small cohort demonstrated significant and sustained B-cell depletion with some clinical benefit [262]. A subsequent multicentre placebo controlled RCT confirmed clinical efficacy and safety and further analysis is underway [263].

##### Iscalimab (also known as ZF-533)

Iscalimab is a fully humanized anti-CD40 monoclonal antibody that blocks CD40. In a phase II placebo-controlled RCT of 44 individuals, iscalimab was shown to be safe and well tolerated with a measurable biological effect on germinal centre formation and improvements in the ESSDAI and ESSPRI in the treated cohort [264].

##### JAK and BTK inhibitors

JAK inhibition suppressed expression of IFN-related genes and BAFF in both a mouse model of SD and human salivary gland epithelial cells *in vitro* [265]. There are a number of studies underway looking at JAK inhibitors in SD but none has reported clinical benefit to date.

Bruton Tyrosine Kinase (BTK) is a cytoplasmic tyrosine kinase and a member of the Tyosine-protein kinase (TEC) family. It is selectively expressed on cells of both the adaptive and innate immune system including B cells, macrophages, thrombocytes, mast cells and basophils. BTK inhibition has been shown to be effective in B-cell malignancies [266] and interest is growing in its potential use in B-cell driven autoimmune diseases [267]. LOU064 is a novel covalent BTK inhibitor that has shown *in vitro* selectivity against relevant kinases with high potency and efficacy in preclinical models of inflammation [268] and preliminary reports from the phase II/III clinical trials suggest a favourable safety profile and some improvement in ESSDAI, salivary flow rates and immunoglobulins [269].

##### Anti-ICOS Ligand mAb

MEDI5872, a fully humanized Anti-ICOS Ligand mAb, interferes with inflammatory pathways by binding to ICOSL [270]. In a small placebo-controlled phase II RCT a reduction in RF levels was noted in the treatment group, but no change was seen in clinical parameters [270].

There were similar findings with a cathepsin S inhibitor [271] which in a double-blind RCT in 75 individuals reduced RF and immunoglobulin levels in the treated group over 12 weeks. There was no demonstrable change in ESSDAI or ESSPRI, so it is not being further developed.

##### Tocilizumab

There were initial case reports of individuals with SD responding to treatment with tocilizumab with improvement in salivary and lacrimal flow rates and reduction of inflammatory infiltrates on minor salivary gland biopsy in one case [272] and improvement in SD-associated myelitis in another [273]. A subsequent multicentre, double blind RCT of 110 individuals failed to show any clinical advantage of tocilizumab compared with placebo over a 6-month study period [274]. *Post hoc* assessment of trial data from the ETAP trial showed that CRESS response rates at the primary endpoint visits were 18% (10 of 55) for tocilizumab *vs* 24% (13 of 55) for placebo (*P* = 0.48) in the ETAP trial.

##### Rituximab

An initial open label study of rituximab in a small cohort of those with early SD confirmed effective B-cell depletion and appeared to demonstrate clinical improvement, especially in those with residual glandular function [275]. This was followed by a flurry of case reports and small case series reporting successful treatment of systemic complications including lymphoma, immune thrombocytopaenia, cryoglobulinaemia, lung disease, membranoproliferative glomerulonephritis and neurological disease in SD [275–286]. Two small RCTs over 24 and 48 weeks suggested beneficial effects on fatigue [287] and salivary flow rates [288]. However, neither of the subsequent larger phase III placebo-controlled trials reached their primary endpoint [289, 290] evaluating patient-reported improvements in pain, fatigue and dryness. The TEARS study included 120 individuals with active disease randomized to either two infusions of rituximab 2 weeks apart or placebo [289]. This study failed to achieve a significant improvement in VAS measures of dryness, global disease activity, fatigue and pain, despite an improvement in salivary flow rates and a measurable laboratory response. The TRACTISS trial of 133 individuals gave two infusions of rituximab at baseline and repeated at 6 months [290]. Again, there were no significant improvements in outcomes overall although the authors noted a small improvement in unstimulated salivary flow rates. However, *post hoc* assessment of the TRACTISS trial data showed that CRESS response rates at the primary endpoint visits were 49% (33 of 67) for rituximab *vs* 30% (20 of 66) for placebo (*P* = 0.026).

Two systematic reviews and a meta-analysis of rituximab treatment for SD [291, 292] concluded that although there was some weak evidence of an improvement in lacrimal gland function there was no overall evidence of improvement in oral dryness, fatigue or QoL, and insufficient evidence to support routine use. There is some evidence, however, that it may have a role to play in those with specific organ manifestations including ILD [293]. The North American guideline group concluded that there was sufficient evidence to suggest rituximab when conventional therapies, including immunomodulators, had proven insufficient. They recommended that it was considered for those with a range of systemic complications including vasculitis, severe parotid swelling, inflammatory arthritis, pulmonary disease and peripheral neuropathy [207, 208]. The most recent European guidelines have suggested that rituximab may be considered for severe, refractory systemic disease, especially those with cryoglobulinaemic vasculitis [257].

Rituximab has also been commonly prescribed by paediatricians for selected jSD cases, with 40% of the surveyed clinicians stating that they have used it for systemic manifestations and 9% for recurrent parotitis [258]. Rituximab has also been found to be beneficial in treating MALT lymphoma and neurological manifestations in children as per various case reports [209].

##### RSLV-132

RSLV-132 is a fusion protein comprising RNase1 fused to the Fc region of IgG1. It promotes digestion of RNA-associated immune complexes reducing Toll-like receptor (TLR) activation with the objective of reducing type 1 IFN, B-cell activation and autoantibody production. In a phase II study in SD, RSLV-132 appeared safe and well tolerated. There was no mandated ESSDAI entry criteria so the study was not powered to indicate an ESSDAI change but there did appear to be a reduction in both physical and mental fatigue in the treatment group [294].

#### Recommendation

Biologic drugs are not currently recommended for use in SD outside of the treatment of specific systemic complications (2, C) (SOA 93.5%).

#### Treatment of systemic disease – miscellaneousIVIG

There is anecdotal evidence supporting the use of IVIG therapy in SD-associated sensorimotor and non-ataxic sensory neuropathy from retrospective and observational cohorts and case reports [295, 296]. Immunoglobulin treatment has also been used successfully in refractory SD-associated myositis not responding to conventional treatment [117]. There is no evidence for its routine use in those without significant systemic disease. It is expensive and not without potential safety concerns.

##### Recommendation

Intravenous immunoglobulins are not routinely recommended for use in SD outside of the treatment of specific systemic complications (2, C) (SOA 96.9%).

##### Colchicine

There are case reports describing successful treatment of SD-associated hypergammaglobulinaemic purpura [297], non-cryoglobulinaemic vasculitis [298], granulomatous panniculitis [299] and pericarditis [300] with colchicine. It is generally safe and well tolerated.

##### Recommendation

Colchicine may be helpful in SD presenting with specific systemic complications (2, C) (SOA 91.4%).

### 10b. What treatments are beneficial for recurrent parotitis in jSD?

Recurrent, treatment resistant parotitis can be a particular problem in jSD. A systematic review of the management of juvenile recurrent parotitis (not SD specific) [301] found 24 relevant studies, of which only one was a RCT. They concluded that the available evidence was weak and difficult to interpret because of the lack of RCTs, the heterogeneity of the definitions used and the high rate of spontaneous resolution.

A case series of six boys with parotitis (not SD related) [302] showed a benefit of saline irrigation of the gland with total resolution of symptoms in two and improvement in four.

A survey of 135 paediatricians treating jSD reported use of various therapies for management of recurrent parotitis: HCQ (65%), CS (57%), MTX (42%), MMF (10%), rituximab (9%), abatacept and AZA (2%), and belimumab (1%) [258].

### Recommendation

Treatment of parotitis in jSD (once infection and stone disease have been excluded) could include the following escalating therapies. A short course of NSAIDs or oral steroids combined with massage followed by washouts with saline or steroids. Consider anti-B-cell-targeted therapies in selected, refractory cases (2, C) (SOA 91%).

### 11. In people with SD, is early treatment of hypergammaglobulinaemia or systemic disease more effective than delayed treatment at slowing disease progression?

The KISS cohort study [198] followed 256 individuals with SD over 3 years. They found an association between persistent hypergammaglobulinemia, falling salivary flow (*P* = 0.008) and solid organ damage (*P* = 0.039) over time. Conversely, those in whom IgG level fell showed less organ damage over time. They assessed organ damage as neurological or pleuropulmonary damage, renal impairment or lymphoproliferative disease. The use of HCQ was associated with less solid organ damage (*P* = 0.008). Overall numbers were low and the length of follow-up (3 years) may be inadequate to reflect longer term outcomes, but the authors concluded that monitoring of IgG levels was helpful in predicting outcomes and suggested that hypergammaglobulinemia was a candidate target to direct treatment.

The presence of hypergammaglobulinemia and hypocomplementemia have been shown to predict progression to SD over time in a cohort of individuals with some features of SD but failing to meet diagnostic criteria at baseline [303].

A multicentre retrospective study of 221 individuals with SD, of whom 77% were exposed to HCQ, evaluated the development of extraglandular manifestations over time and correlated this with HCQ use [199]. They found lower prevalence of arthritis, fatigue, purpura, Raynaud’s and hypergammaglobulinemia in the treated group over time.

### Recommendation

In SD with significant hypergammaglobulinemia consider a trial of HCQ for 6–12 months (2, C) (SOA 94.2%).

### 12. What are the recommended therapeutic options in individuals with SD overlapping with other rheumatic diseases, for example, RA, SLE or scleroderma?

A number of conditions are commonly found in association with SD but the literature on management of these overlaps is scanty and mostly based on anecdotal reports.

#### Multiple sclerosis and SD

There are potentially overlaps in susceptibility genes and mechanisms of disease between SD and multiple sclerosis (MS) with the JAK-STAT signalling pathways playing a role in both, leading researchers to suggest JAK-STAT inhibitors as potential therapies for both MS and SD [304].

#### RA and SD

A single-centre study found that of its 1100 individuals with RA, 12% had RA/SD overlap and were less likely to achieve US remission of their inflammatory joint disease [305].

#### SLE and SD

SD/SLE overlap is common, with one study estimating it affects roughly 23% with an incident diagnosis of SLE [306]. The frequency of overlap increases with age. Those with overlap were more likely to have raised serum levels of pro-inflammatory cytokines, leukopenia and peripheral neuropathy, and less likely to have renal involvement. Treatment should depend on the level of organ involvement and be directed by clinical findings.

#### Scleroderma and SD

A two-centre retrospective observational study included 534 individuals with scleroderma, of whom 14 had overlap with SD [307]. This latter group had higher overall mortality and were more likely to receive immunomodulatory drugs.

Data from the UKPSSR [97] showed that, among 549 subjects where an extensive autoantibody profile was available, ACA was present in 1.3% and anti-Scl70 antibody was present in 1.5% [98]. In a Japanese cohort, 15.6% of the anti-Ro/La negative individuals with SD were ACA positive [308].

### Recommendation

In individuals with overlap CTDs take all confirmed disease entities into account when planning investigation and management (2, C) (SOA 96.3%).

### 13. In people with SD, what is the clinical effectiveness of nutraceuticals in the management of the condition?

Nutraceuticals are products derived from food sources that claim nutritional and/or health benefits. A 2021 review of the current literature on vitamin supplementation in dry-eye disease [309] found that in those with vitamin A deficiency systemic supplementation was effective in treating ocular surface disease, leading to a reduction in dry-eye signs and symptoms. Local (topical) application of vitamin A is also effective in reducing signs and symptoms of dry-eye disease with seven controlled studies all showing benefits to the vitamin A preparation over the comparator. Several of the commercially available eye ointments contain vitamin A.

In a single-centre observational study individuals with sicca were asked to complete a self-assessment questionnaire on diet pre-symptom onset [310]. Adherence to a Mediterranean diet was associated with a lower likelihood of having SD.

A systematic review and meta-analysis of levels of oxidative stress markers and antioxidants in dry-eye disease included nine articles and found an overall increase in oxidative stress markers in dry-eye disease compared with healthy controls [311].

The evidence for omega-3 supplementation is conflicting. A study of 108 individuals with SD and 100 healthy controls evaluated omega-3 and omega-6 intake and serum levels [312]. They found lower levels of omega-3 and omega-6 intake in the SD cohort but poor correlation with serum levels. Lower ocular symptoms, ESSDAI scores and salivary chemokine (C-C motif) ligand 2 (CCL2) correlated with higher omega-3 levels [312]. A double-blind RCT of high-dose omega-3 supplementation in a total of 535 individuals with dry eye (329 active supplement and 170 placebo) published in 2018 found no significant differences in symptoms or signs after 12 months of treatment [313]. A subsequent Cochrane review [314] of 34 RCTs involving 4314 adults with dry eye suggested a possible role for long-chain omega-3 supplementation in managing dry-eye disease, although the evidence was inconsistent. A meta-analysis of 17 randomized clinical trials in individuals with non-selected dry eye found overall that there was evidence that omega-3 supplementations decreased eye symptoms and corneal fluorescein staining, and increased the TBUT and Schirmer’s test values [315]. The most recently published study (the DREAM study) stratified participants with dry eye into five subtypes, but found that none of the groups demonstrated significant improvement with omega-3 supplementation [316]. Omega-3 supplementation is non-prescribable in the UK but is widely available over the counter.

### Recommendation

Consider vitamin A containing eye ointments (2, C) (SOA 89.8%).

Consider advising omega-3 supplementation in SD (2, C) (SOA 89.8%).

### 14. For people with SD, what cognitive therapy or behavioural change interventions are an effective treatment for fatigue and joint pain?

A systematic review of non-pharmacological interventions for SD [317] identified five studies for review including a total of 130 participants. The majority of the studies were small, of low quality and at high risk of bias. The included studies investigated the effectiveness of an oral lubricating device for dry mouth, acupuncture for dry mouth, lacrimal punctal plugs for dry eyes and psychodynamic group therapy for coping with symptoms. Overall, the studies were of low quality and at high risk of bias. Although one study showed punctal plugs to improve dry eyes, the sample size was relatively small. The authors concluded that further high-quality studies were needed.

A review of interventions to manage fatigue in SD [318] found no evidence to support pharmacological treatment of fatigue. Of the non-pharmacological interventions most studies were small and of relatively poor quality. The authors concluded that based on the few small studies available aerobic exercise appears to be safe and effective.

Transcranial direct current stimulation (tDCS) is a non-invasive method of electrical stimulation of the brain using a weak direct current applied to the scalp through electrodes. A parallel double-blind pilot study of tDCS in 36 females with SD randomized to 20 min sessions for 5 days, demonstrated improvements in both groups but with a significant greater improvement in fatigue severity in the active group *vs* the sham treatment group [319]. There were no differences in sleep quality or pain overall.

Non-invasive vagal nerve stimulation (nVNS) has shown promising results in reducing fatigue in SD. In a pilot study [320], 15 subjects with SD used a nVNS device twice daily over a 26-day period and showed significant reduction in fatigue and daytime sleepiness. A recent sham-controlled study in 40 participants with SD showed significant improvements in three measures of fatigue at day 56 [321], suggesting that further larger studies may be worthwhile.

### Recommendation

We recommend an individualized holistic review for those with fatigue focusing on activity management (for example planning, prioritizing, pacing), sleep quality and lifestyle (2, C) (SOA 96.7%).

### 15. In people with SD, what type and frequency of exercise is an effective treatment for fatigue?

An RCT of supervised resistance exercise over 16 weeks conducted in 51 volunteers with SD (26 allocated to exercise group) showed improvements in functional capacity as measured by the Fullerton functional fitness test and the physical (but not emotional) domains of the Short Form Health Survey (SF-36) [322]. There was no change in the ESSDAI.

A supervised walking programme in a small group with SD (23 *vs* 23 non-active controls) demonstrated improved cardio-respiratory fitness with improvement in fatigue scores, reduced depression and improvements in the physical and mental components of the SF-36 [35, 323].

A single-blind randomized pilot study of resistance exercise in 59 females with SD found that the exercise improved symptoms of fatigue and pain but had no effect on disease activity [324]. VAS for pain and fatigue showed significant improvement in the exercise group as did the Functional Assessment of Chronic Illness Therapy (FACIT) score. There was no change in ESSDAI. The ESSPRI showed significant improvement in pain and fatigue but no change in dryness.

A randomized trial of cardiovascular exercise in a group of 60 females with SD confirmed improvement in maximal oxygen uptake (VO2max, a measure of maximal aerobic capacity) and anaerobic threshold in the exercise group with 28 completing the exercise protocol [324]. ESSDAI remained stable in both groups. The SF-36 questionnaire improved in both groups with no difference between the groups.

In an unblinded, uncontrolled pilot study 23 volunteers with SD were enrolled into 60-min Pilates classes, three times a week for 8 weeks. No detail was provided in the results but the authors report statistically significant improvements in measures of fitness, mobility and emotional health [325].

There are also data from other chronic conditions that may potentially be extrapolated to SD, e.g. adding in activity pacing to an exercise intervention in 21 people with MS helped improve activity levels without exacerbating fatigue [326]. In a narrative review, the authors discussed the potential of activity pacing to increase physical activity and lessen fatigue in individuals with disabling conditions [327].

Overall exercise appears beneficial for fatigue in SD but there is insufficient evidence to recommend one type of exercise over another.

### Recommendation

Exercise is safe and potentially beneficial for those with SD and fatigue (2, C) (SOA 97.9%).

### 16. For pregnant people with SD, both with and without anti-Ro and/or La antibodies, is HCQ and/or low-dose aspirin effective in reducing fetal mortality and morbidity?

The presence of anti-Ro and/or anti-La antibodies in the maternal circulation are associated with congenital fetal heart block (CHB) and congenital neonatal lupus rash (cNL) [328], with studies suggesting that CHB prevalence is higher in those with high-titre antibodies [329] and those who are positive for both antibodies [330], whilst cNL is higher in female children and those exposed to anti-La antibody [328]. Recurrence rates of CHB are significantly higher in subsequent pregnancies following an index CHB case [331]. A systematic review of a total of 16 case–controlled and observational studies representing 1706 anti-Ro antibody positive and 454 anti-La antibody positive females reported a prevalence of 1.8% for CHB but were not able to determine whether this was modified by being on HCQ or not [332]. However, a multicentre, open-label clinical trial (PATCH) involving 54 women who had had a previous CHB foetus showed that HCQ 400 mg daily reduced the prevalence of recurrence to below 50% of expected [333]. Furthermore, a multicentre case–control study involving 556 children born to anti-Ro and or anti-La antibody positive mothers with an underlying rheumatological disease found that exposure to HCQ was associated with a reduced risk of cNL [328]. HCQ and low-dose aspirin have both been shown to be safe in pregnancy [334].

Clinical practice in the UK varies. Many units offer pre-pregnancy counselling to discuss the risks. Many units routinely recommend aspirin from 12 weeks of pregnancy, based on the evidence from systematic reviews that it reduces the risk of pre-eclampsia [335]. Some offer HCQ to those who are anti-Ro antibody positive on the basis of the risk reduction seen in the PATCH study [333].

### Recommendations

Recommend low dose aspirin if high risk of pre-eclampsia or high-risk pregnancy in general (1, A) (SOA 93.8%).

Consider HCQ during pregnancy for those who are anti-Ro antibody positive on the basis of the risk reduction seen in the PATCH study (2, C) (SOA 91.5%).

Offer HCQ in subsequent pregnancies to those who have experienced CHB in a previous pregnancy (1, B) (SOA 96.7%).

### 17. For pregnant people with SD, with a fetus who has an incomplete heart block or hydropic changes, are fluorinated steroids and/or immunoglobulins effective in decreasing the likelihood of congenital heart block in the fetus?

Case reports and small case series [336] have shown that both plasmapheresis and immunoglobulins reduce circulating anti-Ro antibody levels in the maternal circulation, and it was postulated that these treatments might lower the risk of CHB in high-risk pregnancies. Ruffatti *et al.* [337] prospectively treated 12 mothers with CHB fetuses with weekly plasmapheresis, fortnightly IVIG and daily betamethasone 4 mg from detection of CHB until delivery. Of the six with second-degree block, one reverted to normal atrio-ventricular conduction and two to first-degree block following treatment, three continued with second-degree block but did not progress. The six with third-degree block showed no response to treatment, and three of these subsequently required pacemakers. All 12 children survived. A systematic review and meta-analysis of the use of antenatal fluorinated CS to prevent CHB included a total of 12 studies and concluded that fluorinated steroids did not provide a significant benefit in fetuses with CHB [338].

A single-centre review of 59 cases of CHB compared 29 treated with 8 mg dexamethasone per day at <24 weeks gestation with 30 treated with either 4 mg per day or started at >24 weeks gestation [339]. They found that CHB resolved in 5 of the 29 treated early with 8 mg compared with none in the comparator group. However, CHB reappeared in all 5 either pre- or post-natally.

Current UK practice varies but some units, e.g. experts from Great Ormond Street Hospital are treating with dexamethasone once CHB is detected. There is currently no international consensus on best practice.

### Recommendation

Refer urgently to specialist centre if CHB is detected for consideration of treatment with dexamethasone (2, C) (SOA 98.9%).

### 18. In people with SD, what is the most clinically effective long-term follow-up programme and how should this be personalized?

There is little evidence in the literature regarding optimum long-term follow-up of SD.

A single-centre long-term follow-up study of a cohort of people with undifferentiated CTD found that 3% per annum developed a definite CTD and were more likely to do so if they had a positive ENA [340]. Two evolved into SD and both were anti-Ro antibody positive at baseline.

A retrospective follow-up study of a population of 967 individuals with SD found that men were more likely to develop ILD, lymphadenopathy and lymphoma, whilst women were more likely to develop hypothyroidism over time [341].

There is poor consensus on appropriate frequency of follow-up for patients with SD and we would recommend that this is determined on a case-by-case basis taking into account length of diagnosis, number of risk factors for lymphoma development, presence of extraglandular disease and whether they are on immunosuppressive drugs. Appropriate ongoing investigations should be arranged as appropriate, e.g. lung function tests should be organized for those with documented lung disease at annual intervals or sooner if clinically indicated.

### Recommendations

Consider follow-up within Rheumatology for those with confirmed SD, particularly if there is evidence of systemic disease (2, C) (SOA 91.9%).

### 19. What age-tailored information, education and support do people with SD and their families and carers need and how can they access this?

Analysis of a comprehensive survey of individuals with SD undertaken by the USA based charity—the Sjogren’s Foundation—found that the most frequent extraglandular symptoms included fatigue, dry/itchy skin and morning stiffness [342]. They found a high burden of disease and identified that the top three symptoms or signs that individuals with SD hope new treatments will address are dryness, fatigue, and reduction in lymphoma or blood cancer risk.

The high symptom burden was confirmed in a qualitative study which included moderated online discussion forums and one-to-one questionnaires [343]. In this study fatigue was rated as the most severe and burdensome symptom.

Significant unmet needs have been identified within Europe for those with SD and their families/carers [344] and efforts are underway to address this.

A qualitative focus group study involving individuals with SD and their spouses [3] found that they wanted tailored support from healthcare professionals, including information provision, access to peer support and professional support. The authors proposed a three-step model of care comprising written information, education groups, peer support, digital self-management and one-to-one therapy.

In a study of 98 women with SD those who demonstrated adaptive coping strategies had better sexual function and lower levels of sexual distress than those with maladaptive coping strategies and the authors suggested that the development of psychosocial or interpersonal interventions for individuals with SD were warranted [14].

There has been work in the UK to develop a non-pharmacological intervention model to improve QoL in SD [345].

A review of the resources available on YouTube for SD [346] found approximately half of the videos (51.4%) to be useful, with 8.6% providing misleading content. The authors concluded that people should be directed towards validated resources and that specialists should actively participate in the development of video-sharing platforms.

Patients benefit from a holistic review taking into account their ocular, oral and systemic symptoms and addressing their individual needs.

### Recommendation

Provide written information on the manifestations of SD and their management, direct individuals with SD to appropriate online resources and recommend they access local and national support groups, e.g. Sjogren’s UK Home—Sjögren’s UK (sjogrensuk.org), Sjogren’s Foundation (www.sjogrens.org), Versus Arthritis and NHS websites (2, C) (SOA 97.1%).

## Applicability and utility

The final guideline will be disseminated by publication in the journal *Rheumatology (Oxford)* and will be freely available on the BSR website.

It is recognized that constraints within the healthcare system may create challenges to widespread implementation of this guideline. For instance, many centres do not have access to minor salivary gland biopsy and not all have access to expert salivary gland USS. Access to certain treatments, e.g. serum eye drops are limited by cost and availability and there are currently no immunomodulatory treatments licensed for use in SD. Most of the immunosuppressive drugs are used off-licence for this indication. Biologics are not NICE approved for SD. Of the few patients who do get biologics this is usually either as part of a clinical trial or because they meet criteria for RA or another CTD (usually SLE).

## Research recommendations

There are significant unmet needs in the management of this patient cohort. Further research into pathogenetic mechanisms may facilitate the development of targeted treatments. Accurate stratification of patients into disease subgroups and collaborative studies are essential to provide large enough cohorts to demonstrate meaningful effects of interventions. There is a need to develop better measures of disease activity as the currently used parameters do not include fatigue and dryness, underestimate the disease burden and are not sensitive to change. There is also a need to develop Quality Standards for SD to improve standards of care.

## Audit

A model audit tool is available via the BSR website and in Supplementary Data S2 available at *Rheumatology* online. We would also strongly recommend that new cases of SD are recorded in the NEIAA (New Early Inflammatory Arthritis Audit https://arthritisaudit.org.uk/) database to provide information on the incidence and demographics of the condition plus collect evidence on diagnostic delays and route of referral.

## Conclusions

SD remains an under-recognized condition with significant unmet needs. Nonetheless, we do feel that following these guidelines will provide a framework for health professionals to manage those with SD effectively and proactively. There are a number of studies underway investigating non-pharmacological treatments, novel biologic drugs and repurposing of existing conventional and biologic immunosuppressive agents. The NEIAA has recently expanded to include new CTD diagnosis, including SD, and we would encourage teams to record all newly diagnosed cases.

## Supplementary Material

keae152_Supplementary_Data

## Data Availability

Data are available in the guideline and its supplementary material.
