# Supplementary material for: British Society for Rheumatology guideline on management of adult and juvenile onset Sjögren disease
Source: Rheumatology (Oxford). 2024 Apr 16;64(2):409–39. doi: 10.1093/rheumatology/keae152 (PMC12013823; doi:10.1093/rheumatology/keae152)
Supplement: keae152_Supplementary_Data [file keae152_supplementary_data.zip › rhe-23-2396-File006.docx]

**British Society for Rheumatology guideline on management of adult and juvenile onset Sjogren Disease (SD) 2024**

**Recommended audit tool**

Based on the BSR management guidelines

| **Recommendation** | **Yes** | **No** | **Not**  **applicable** | **Comments** |
| --- | --- | --- | --- | --- |
| Has the diagnosis of SD been made in line with 2016 ACR/EULAR criteria (1):   - Anti-Ro antibodies (3) - Focus score of > or =1 (3) - Abnormal ocular staining score > or =5 (1) - Schirmer’s test result of < or = 5mm/5 min (1) - Unstimulated salivary flow <0.1ml/min (1)   For classification as SD require score > or = 4 |  |  |  |  |
| Have you entered case into the NEIAA if newly diagnosed? |  |  |  |  |
| **Diagnosis** |  |  |  |  |
| Have you checked both ANA and ENA? |  |  |  |  |
| Have you done an Ultrasound of the salivary glands? |  |  |  |  |
| Have you organised a minor labial salivary gland biopsy to aid diagnosis and/or inform prognosis? |  |  |  |  |
| **Management** |  |  |  |  |
| Have you checked for new salivary gland swelling or other symptoms that might suggest the development of lymphoma and offered investigation if appropriate? |  |  |  |  |
| Baseline assessment – have you done:   - Clinical exam - Routine bloods (FBC, U&E/LFT) - Immunoglobulins - C3/C4 - TFT - TTG - CK - Serum bicarb - Vitamin D |  |  |  |  |
| **Ocular management** |  |  |  |  |
| Have you recommended a preservative free moisturising eye drop a minimum of four times daily? |  |  |  |  |
| Have you recommended a heated eye compress for at least 10 minutes daily? |  |  |  |  |
| Have you considered lipid containing eye drops or liposomal eye sprays as adjunctive treatment? |  |  |  |  |
| If persistent symptomatic dry eye despite above have you considered referral to ophthalmology to consider punctal plugging, antibiotics, immunosuppressive or serum eye drops? |  |  |  |  |
| **Oral management** |  |  |  |  |
| Have you suggested saliva substitutes for symptomatic relief of oral dryness? |  |  |  |  |
| Have you recommended regular brushing with fluoride toothpaste, proactive dental care and the use of xylitol containing products to prevent dental decay? |  |  |  |  |
| **Systemic management** |  |  |  |  |
| Have you considered a trial of pilocarpine (5mg once daily increasing to 5 mg tds/qds) in those with significant ocular and/or oral sicca symptoms? |  |  |  |  |
| Have you considered topical oestrogen creams/pessaries and/or non-hormonal vaginal moisturisers in peri- or post-menopausal women with significant vaginal dryness ? |  |  |  |  |
|  |  |  |  |  |
| Have you considered a trial of Hydroxychloroquine for 6 to 12 months in patients with significant fatigue and systemic symptoms or hypergammaglobulinaemia |  |  |  |  |
| Have you prescribed steroids and if so for what indication? |  |  |  |  |
| Have you prescribed cDMARDS, biologics or immunoglobulins and if so for what indication? |  |  |  |  |
| Have you prescribed colchicine and if so for what indication? |  |  |  |  |
| Is there recurrent parotitis and if so have you offered appropriate management? |  |  |  |  |
| Does the individual have another connective tissue diseases and has this been taken into account when planning investigation and management? |  |  | ` |  |
| Have you considered a trial of vitamin A containing eye ointments and omega-3 supplementation? |  |  |  |  |
| Have you asked about fatigue and discussed suitable management strategies including recommending exercise? |  |  |  |  |
| Have you counselled on pregnancy risks and/or referred for specialist advice if appropriate? |  |  |  |  |
| Have you booked an appropriate follow-up appointment? |  |  |  |  |
| Have you provided written information, directed to appropriate on-line resources and recommended that they access local and national support groups? |  |  |  |  |

Abbreviations: SD sjogren disease; ACR/EULAR American College of Rheumatology/European League against Rheumatic diseases; ANA anti-nuclear antibodies; ENA extractable nuclear antigens; FBC full blood count; U&E urea and electrolytes; C3/C4 complement 3/ complement 4; TFT thyroid function; TTG tissue transglutaminase; bicarb bicarbonate; cDMARDS conventional disease modifying anti-rheumatic drugs;

1. Shiboski CH, Shiboski SC, Seror R, Criswell LA, Labetoulle M, Lietman TM, et al. 2016 American College of Rheumatology/European League Against Rheumatism Classification Criteria for Primary Sjogren's Syndrome: A Consensus and Data-Driven Methodology Involving Three International Patient Cohorts. Arthritis Rheumatol. 2017;69(1):35-45.
